# Conformational Changes in Acetylcholine Binding Protein Investigated by Temperature Accelerated Molecular Dynamics

**DOI:** 10.1371/journal.pone.0088555

**Published:** 2014-02-13

**Authors:** Zeynab Mohammad Hosseini Naveh, Therese E. Malliavin, Luca Maragliano, Grazia Cottone, Giovanni Ciccotti

**Affiliations:** 1 School of Physics, University College Dublin, Dublin, Ireland; 2 Institut Pasteur and CNRS UMR 3528, Unité de Bioinformatique Structurale, Paris, France; 3 Department of Neuroscience and Brain Technologies, Istituto Italiano di Tecnologia, Genoa, Italy; 4 Department of Physics and Chemistry, University of Palermo, Palermo, Italy; 5 Department of Physics, University of Roma “La Sapienza”, Rome, Italy; Wake Forest University, United States of America

## Abstract

Despite the large number of studies available on nicotinic acetylcholine receptors, a complete account of the mechanistic aspects of their gating transition in response to ligand binding still remains elusive. As a first step toward dissecting the transition mechanism by accelerated sampling techniques, we study the ligand-induced conformational changes of the acetylcholine binding protein (AChBP), a widely accepted model for the full receptor extracellular domain. Using unbiased Molecular Dynamics (MD) and Temperature Accelerated Molecular Dynamics (TAMD) simulations we investigate the AChBP transition between the apo and the agonist-bound state. In long standard MD simulations, both conformations of the native protein are stable, while the agonist-bound structure evolves toward the apo one if the orientation of few key sidechains in the orthosteric cavity is modified. Conversely, TAMD simulations initiated from the native conformations are able to produce the spontaneous transition. With respect to the modified conformations, TAMD accelerates the transition by at least a factor 10. The analysis of some specific residue-residue interactions points out that the transition mechanism is based on the disruption/formation of few key hydrogen bonds. Finally, while early events of ligand dissociation are observed already in standard MD, TAMD accelerates the ligand detachment and, at the highest TAMD effective temperature, it is able to produce a complete dissociation path in one AChBP subunit.

## Introduction

The transmembrane nicotinic acetylcholine receptors (nAChRs), belonging to the so-called ‘Cys-loop’ super-family of ligand-gated ion channels (LGICs) [1], [2], are involved in a variety of biological processes [3]–[5] and have been implicated in the onset of Alzheimer’s disease [4], [6] and nicotine addiction [7]. The nAChR channel pore, located in the transmembrane domain (TMD), opens following the binding of agonist ligands in the orthosteric site located in the extracellular domain (ECD) of the protein. Conversely, the channel is mainly closed in the resting state, when either no ligand is present or an antagonist is bound, and in the desensitized, agonist-bound state. The main endogenous agonist for nAChRs is acetylcholine (ACh). Nicotine also binds nAChR as an agonist while lobeline (Fig. S1) has a full or a partial agonist effect [8], [9].

**Figure 1:**
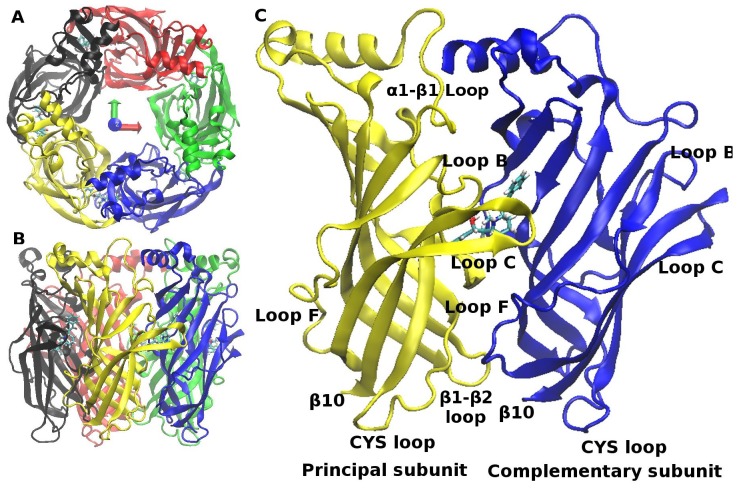
The structure of AChBP. The five homomeric subunits in AChBP are labeled S1, S2, S3, S4, S5, in a clockwise direction as viewed from the apical side and colored black, red, green, blue, yellow respectively. A) View from the apical side of the AChBP protein, shown as a cartoon model; B) Side view; C) The AChBP subunit interface bound to the ligand lobeline, represented in sticks. The relevant loops on both the principal and complementary subunit are indicated.

Currently, only limited information is available on the atomic structure of nAChRs. Low resolution structures of *Torpedo* acetylcholine receptor with closed [10] and open [11] pore were obtained from electron microscopy data. Structures of distant prokaryotic homologues of nAChRs, present in *Gloeobacter violaceus* and in *Erwinia chrysamthemi*, GLIC and ELIC, were solved during the last years by high-resolution crystallography [12]–[17], [17]–[19]. Interestingly, the GLIC structures put in evidence a binding site for anesthetics in the TMD [20], ketamine binding to the orthosteric site [21] as well as a new semi-closed state of the channel [22]. The ELIC structures showed the binding of anesthetics both in the TMD and in the ECD [23] and the binding of acetylcholine to the ECD [21]. However, despite these new pieces of information, the nAChR gating conformational transition is not yet fully understood. Valuable insight on the ligand binding mechanism came from studies of a water-soluble homologue of the nAChRs ECD, the pentameric acetylcholine binding protein (AChBP). Indeed, AChBPs have been crystallized bound to different ligands displaying agonist or antagonist effects on nAChRs [24], [25]. The structures of unliganded (apo) [26], [27] and liganded (holo) state of AChBP [27]–[40] revealed how the ligands bind to the orthosteric pocket. This pocket, present in each of the subunits, is lined by the so-called loop C and extends at the interface between subunits (Fig. 1). The AChBP structures revealed the influence of the ligand type on the degree of C-loop closure against the protein core [32], as the loop arrangements cluster into three groups: i) the antagonist-bound “open” conformation; ii) the apo “intermediate” conformations; iii) the agonist-bound “closed” conformations. It is worth noting however that a recent AChBP structure [41] questions this model, in the case of the small antagonist ligand dihydro-beta-erythroidine (DH
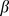
E).

Although the ECD/TMD interface region [31], involved in discriminating between agonist versus antagonist binding and in the long-range communication between the extracellular binding site and the transmembrane pore, is not present in AChBP, similar structural patterns and pharmacological responses have made AChBP a valuable proxy for investigating ligand recognition by nAChRs.

Due to the lack of high-resolution structural information on the nAChRs, computational methods have been extensively used to probe the conformational transition. Nanosecond time scale Molecular Dynamics (MD) simulations have been performed on homology models of nAChRs [42]–[44]. Along with normal mode analysis, performed by using an elastic-network representation for the protein [45], [46] or on a full atomistic protein model [44], [47], [48], they provided first useful insights into the gating mechanism. However, because the binding-to-gating process in nAChRs takes place in the millisecond timescale in physiological conditions [49], standard MD approaches are not useful. Targeted MD has been used, in which the C-loops have been forced to move from the outward to the inward, agonist bound, position [50], [51]. 10 ns MD simulations have been performed on AChBP in complex with acetylcholine [52], providing a picture on the ligand interaction with binding site residues; with nicotine and carbamylcholine [53], focusing mostly on the dynamics of residues and water in the binding pocket rather than on large global motions, and with partial agonists [54]. Spontaneous, although preliminary, conformational changes have been observed in unbiased MD simulation of a homology model of the 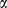
7 nAChR ECD both in the apo state and bound to an antagonist toxin [55]. Steered MD was used to study the unbinding of nicotine from the AChBP protein [56]; the unbinding of acetylcholine from a homology model of the ECD of the nicotinic receptor has been investigated, by using as reaction coordinate the distance between the ligand and the binding site [57].

In the present work we use full-atom standard MD and the enhanced sampling method temperature-accelerated MD (TAMD) [58] to study the AChBP holo-to-apo transition. In particular, we investigate the conformational changes involved in the opening and closing of the C-loops. In TAMD, a set of extra variables are introduced, coupled to the original system via collective variables (CVs). The original and new variables are then evolved concurrently in condition of effective adiabatic separation and at two different temperatures, the physiological temperature for the physical system and a higher artificial temperature for the new variables. In this way, the system navigates the free energy landscape associated to the extra variables overcoming barriers that are even much higher than the energy at the physiological temperature. TAMD can be used to reconstruct the free energy landscape from direct sampling via reweighting [58], [59], or combined with a mean-force interpolation method [60]. TAMD has already been applied to a variety of rare events sampling studies [61]–[67] and has proved to be particularly useful in protein conformational searches [68]–[75]. Here, we use it to search for AChBP conformations by exploring the space of a set of suitable variables.

The conformational transition between the lobeline-bound (holo) and the apo states of *Aplysia californica* AChBP is investigated. First, unbiased MD simulations on the hundred of nanoseconds time scale allowed to characterize the relative mobilities of the different AChBP regions and to give an insight on which CVs are suitable to describe the transition between the holo and apo conformations. MD simulations starting from *ad hoc* modified holo conformations put in evidence the influence of few key residues on the nature of the metastability, or in other words, let us to locate the transition bottlenecks at molecular level. Then, TAMD simulations were performed using as CVs the centers of mass of the C-loop and the cys-loop, to accelerate sensibly the holo-to-apo transition of AChBP.

## Results

### MD Simulations

MD simulations of AChBP were carried out for several systems in explicit water, each 150 ns in length: the apo pentamer (P), the lobeline-bound pentamer in the presence (P1+L) or in the absence (P1) of lobeline. Details on the starting structures and simulation methods are given in Section “Materials and Methods” and in Text S1.

As first, an analysis of the protein secondary structure has been performed along the trajectories. The content in 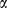 helices and 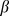 strands, calculated using STRIDE [76], mostly superimpose with the corresponding contents in the X-ray crystallographic structures (data not shown) and is quite similar in all trajectories and subunits except for the two small 3–10 helix (residues 61–63) and 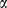-helix (residues 67–70). While the shortest disappears along the simulations, the residue stretch at 67–70 still shows a significant percentage of 3–10 helix content along the whole trajectory in each simulation done. We attribute these secondary structure changes to the absence of crystal packing.

To further assess the stability of the model systems along the trajectories, the Root Mean Square Deviation (RMSD) of the individual subunits with respect to the starting conformations after equilibration was calculated after removing the roto-translational motions of each individual subunit (Fig. S2). All profiles show a plateau after 20 ns, at about 1.2 Å for P1+L and 1.7 Å for P and P1. In P1+L, the RMSD curves of all subunits are superimposed within 0.5 Å, whereas they are superimposed within 1.0 Å and 1.5 Å in P1 and P, respectively: this means that the absence of ligand allows larger conformational changes and more variability among subunits. This is in agreement with results from previous simulations of AChBP [53].

**Figure 2:**
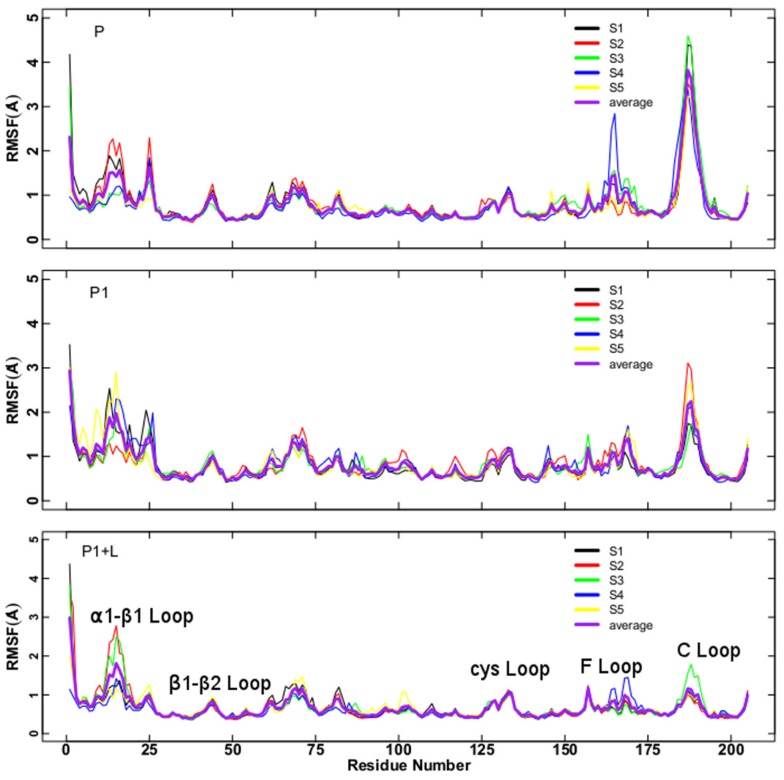
Root Mean Square Fluctuations of C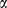 atoms. Root Mean Square Fluctuations (in Å) of C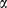 carbon atoms in the individual subunits with respect to the average structure calculated as 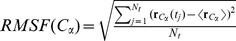, where 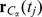 is the position of the 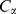 atom at 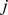
th time step, 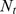 is the total number of steps and 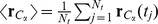, along the the 50–150 ns time interval (i.e. where the RMSDs are stabilized, see Fig. S2). Upper panel: P; central panel: P1; lower panel: P1+L. The curves are colored according to the scheme in Fig. 1. The line in magenta is the average over the five subunits.

The atomic Root Mean Square Fluctuations (RMSFs) profiles (Fig. 2) are similar for the three trajectories P1+L, P1 and P, with the exception of few regions: the C-loop, the F-loop, the 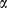
1-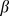
1 loop (residues 9–27) connecting the helix 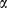
1 and the strand 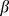
1, and the 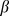
1–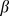
2 loop. With respect to P, in P1+L the RMSFs averaged among subunits are smaller in these regions. In particular, the loop C displays the largest decrease, followed by the loop F. Indeed, because of the interaction with lobeline the loop C is locked in a specific conformation with limited fluctuations [27]. On the other hand, the fluctuations in cys-loop region displays quite similar values in all simulations. Despite the overall similar RMSFs profiles in trajectories P1+L, P1 and P, the differences between P1 and P show that removing ligand does not produce the same RMSFs profile observed for P in the time window considered. The large difference in the C-loop fluctuations is due to the opening of C-loop in P (the distance 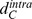, defined in Section “Trajectory Analysis”, is 15.1 Å, averaged over the trajectory and over the five subunits) to a larger extent than in P1+L (averaged 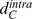 = 13.5 Å) and P1 (averaged 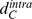 = 13.8 Å). Differences in opening correspond to those observed in the starting X-ray crystallographic structures: 2BYS (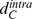 = 12.7 Å, averaged over the five subunits) and 2W8E (averaged 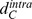 = 14.7 Å).

The cys-loop does not display significant displacement with respect to the remaining part of the pentamer, as the distances 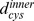 and 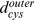 defined in Materials and Methods (Section “Trajectory analysis”) display variations smaller than their corresponding standard deviations (data not shown). This is most likely related to the structure of AChBP, which encompasses only the ECD of nicotinic receptors.

**Figure 3:**
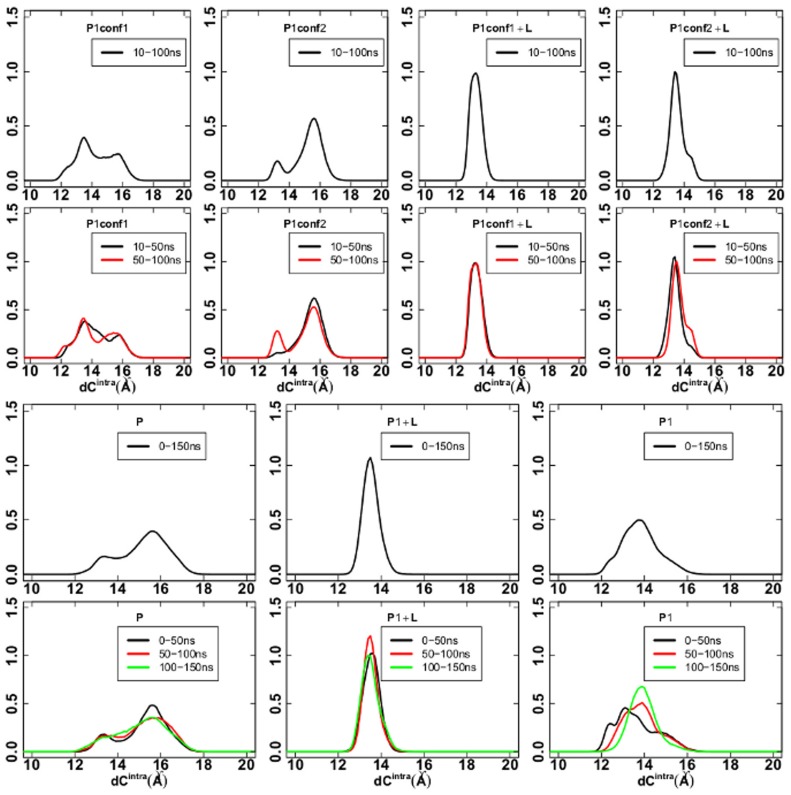
Distribution of 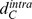 values. First and third rows: distribution of 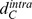 values (Å) calculated over all the MD trajectories; second and fourth rows: distribution of 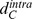 values (Å) calculated in different time intervals (see legends). 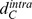 values are collected from the five subunits.

Distributions of 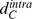 were calculated along the trajectories, by joining together data from all five subunits. Results are shown in Fig. 3. The P1+L distribution is unimodal, centered on the value 13.4 Å, whereas the P distribution is bimodal with two peaks located at 13.2 and 15.6 Å (Fig. 3, first row). The P1 distribution is also uni-modal, although broader than P1+L and displaying a small shoulder at 12.4 Å. The distributions calculated along the three time intervals: 0–50, 50–100 and 100–150 ns (Fig. 3, second row) are almost superimposed for P1+L and P whereas they display more heterogeneity for P1. This is the sign of a reached stable condition of the P1+L and P trajectories. P1 is not yet in stable conditions and this is probably due to the destabilization produced by removing lobeline. This destabilization is not overcome in 150 ns, as is also visible in RMSD and RMSFs plots (see Fig. S2 and Fig. 2).

Starting from biased conformations of AChBP (conf1 and conf2, described in Text S1, section “New AChBP models”) removes this destabilization and makes the 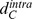 distribution evolve along 100 ns toward the bimodal distribution observed in P (Fig. 3, third and fourth rows). The drift is even more pronounced in P1conf2, where the LYS143 sidechain was modified. At variance, in the presence of lobeline (P1conf1+L, P1conf2+L, see Table 1), the 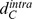 distribution stays unimodal as in the simulation P1+L. The presence of lobeline is thus able to counterbalance the introduced perturbation, even if the orientation of LYS143 is changed.

**Table 1:** Simulations of the AChBP pentamer.

Name	Starting point	Components	Type	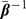 (kcal/mol)	Duration (ns)
P1+L	PDB 2BYS	AChBP, LOB	MD	–	150
P1	PDB 2BYS	AChBP	MD	–	150
P	PDB 2W8E	AChBP	MD	–	150
P1conf1+L	PDB 2BYS 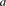	AChBP, LOB	MD	–	100
P1conf2+L	PDB 2BYS 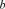	AChBP, LOB	MD	–	100
P1conf1	PDB 2BYS 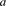	AChBP	MD	–	100
P1conf2	PDB 2BYS 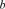	AChBP	MD	–	100
(P1) 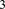	P1 at 40 ns	AChBP	TAMD	3	30
(P1) 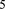	P1 at 40 ns	AChBP	TAMD	5	30
(P1) 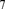	P1 at 40 ns	AChBP	TAMD	7	30
(P1) 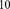	P1 at 40 ns	AChBP	TAMD	10	10
(P1+L) 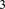	P1+L at 40 ns	AChBP,LOB	TAMD	3	20
(P1+L) 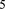	P1+L at 40 ns	AChBP, LOB	TAMD	5	30
(P1+L) 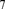	P1+L at 40 ns	AChBP, LOB	TAMD	7	20
(P1+L) 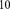	P1+L at 40 ns	AChBP, LOB	TAMD	10	10
(P1-40ns) 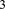	P1+L at 40 ns 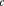	AChBP	TAMD	3	30
(P1-40ns) 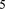	P1+L at 40 ns 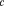	AChBP	TAMD	5	20
(P1-40ns) 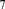	P1+L at 40 ns 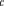	AChBP	TAMD	7	20

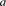
New models (see Section: “New AChBP models” in SM);

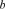
as before, plus disrupting the LYS143/TYR188 bond.

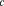
The ligands were removed after 40 ns of the P1+L unbiased simulation. LOB: abbreviation for Lobeline. 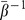 = 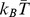 where 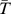 is the artificial temperature.

To summarize, no major change in the average pentamer architecture is observed along the MD trajectories of P1+L, P1 and P. Larger conformational drifts and heterogeneity in atomic fluctuations appear in the absence of lobeline and in the apo system P. The distribution of C-loop openings in P1+L/P1 on one side, and in P on the other side, are respectively unimodal and bimodal, revealing the existence of two metastable states which cannot easily inter-convert in the MD timescale here considered. Biasing the initial sidechain conformations of P1 induces the C-loop opening, in particular if LYS143 is perturbed.

### TAMD Simulations

The data discussed so far confirm that the closed to open transition of the C-loop occurs on very long time scales. To accelerate this transition, three sets of TAMD simulations at four different effective temperatures were carried out using systems all with closed C-loop conformations (see Table 1). The first two sets are for unliganded AChBP and were started from the conformations corresponding to the 40 ns snapshot of the P1 and P1+L free MD simulations, removing the ligand in the second case. We indicate them as (P1)_*i*_ and (P1-40 ns)_*i*_, where 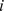 indicates the effective energies 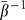 equal to 3, 5, 7 and 10 kcal/mol. The third set is for liganded AChBP, starting from the 40 ns snapshot of the P1+L free MD and is indicated as (P1+L)_*i*_.

As a first check on the TAMD simulations we calculated the protein secondary structure content along the trajectories. Similarly to what was observed along the standard MD, the 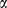 helix and 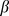 strand content along all TAMD trajectories mostly superimpose with the corresponding contents in X-ray crystallographic structures (data not shown), with the exception of the two small 3–10 helix (residues 61–63) and 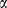-helix (residues 67–70), as already observed along the MD trajectories. This points out that during TAMD the protein secondary structure is not altered, with respect to the MD.

**Figure 4:**
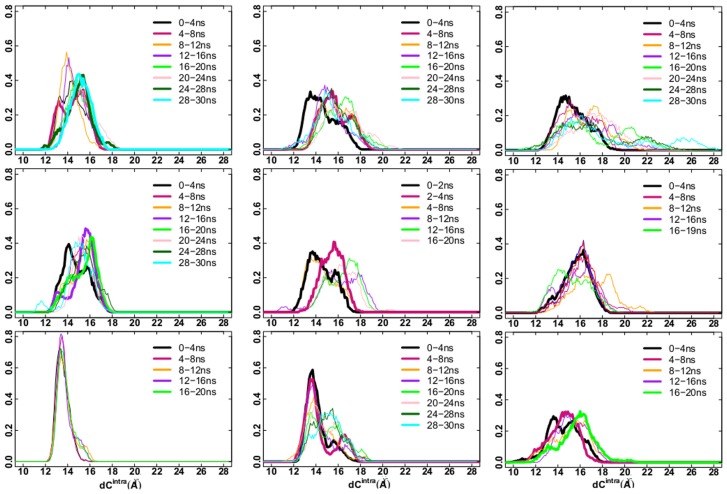
Distribution of 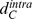 values. Distribution of 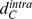 values (Å) calculated over the TAMD trajectory, at different time slices (see legends). Left: 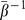 = 3 kcal/mol; central: 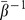 = 5 kcal/mol; right: 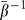 = 7 kcal/mol. From top to bottom: TAMD simulations starting from the P1, P1–40ns and P1+L initial conditions, respectively. 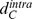 values are collected from the five subunits.

In order to check if TAMD has been successful in driving the C-loop opening, the 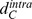 distributions along the TAMD trajectories were calculated and compared with the bimodal distribution obtained in the standard P simulation (see Fig. 3). Results are shown in Fig. 4 for (P1)_*i*_, (P1–40ns)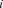 and (P1+L)_*i*_ from top to bottom and 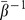 equal to 3, 5 and 7 kcal/mol. Results for 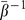 equal 10 kcal/mol are shown in Fig. S3.

It can be observed that in the isolated AChBP system at 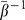 equal to 3 kcal/mol, (P1)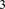, the profile of the 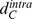 distribution approaches the one for P already in a time scale of few nanoseconds (Fig. 4, left upper panel, red bold line). At later times, the distribution becomes monomodal, but peaked at larger values of 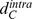 (green and cyan bold lines). Results from TAMD started from the P1-40ns structure also show a bimodal distribution in the interval 0–4 ns (Fig. 4, left middle panel, black bold line); a bimodal distribution even more similar to the one in P is achieved in the interval 12–16 ns (magenta bold line). At later times, the distribution shifts to higher values of 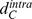 (green bold line). Thus, TAMD accelerates the opening of the C-loops in the unliganded AChBP by at least a factor of 10 with respect to the standard MD simulations, already at 3 kcal/mol.

Conversely, as shown in Fig. 4, left lower panel, the presence of the lobeline prevents this acceleration at the smallest value of 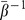, suggesting that a higher effective energy is needed to open the C-loops in the presence of the ligands; indeed, the 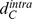 distribution in the presence of lobeline starts to have a bimodal shape (although still with higher percentage of closed C-loops) at 5 kcal/mol, on the 0–4 ns time scale (Fig. 4, central lower panel, black bold line). A secondary small peak at 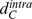 about 17 Å appears later, in the time interval 4–8 ns (red bold line). Increasing the effective energy up to 7 kcal/mol produced an unimodal “open” distribution already before 4 ns (Fig. 4, right upper and middle panels); at variance, in the presence of lobeline, a bimodal distribution is achieved over 0–4 ns (Fig. 4, right lower panel, black curve), with roughly the same percentage of closed and open monomers. Then the distribution switches to unimodal shape over 4–8 ns, with a peak corresponding to an intermediate conformation. A bimodal distribution is observed again, over the last 16–20 ns of TAMD simulation, this time corresponding to mostly open subunits.

An almost wide unimodal distribution is observed over 0–2 ns at 10 kcal/mol in the unliganded case (see Fig. S3, upper panel); in the presence of lobeline, bimodal distribution is achieved over the first 2 ns (Fig. S3, lower panel). Note, however, that, already at 7 kcal/mol, the 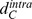 distributions quickly evolve toward a profile much more different than the one observed for P, with 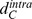 values up to 36 Å. The presence of lobeline is no more an obstacle to the C-loop opening, but TAMD seems to evolve the system to states distinct from the natural apo state P.

### Hydrogen Bonds between AChBP Residues

**Figure 5:**
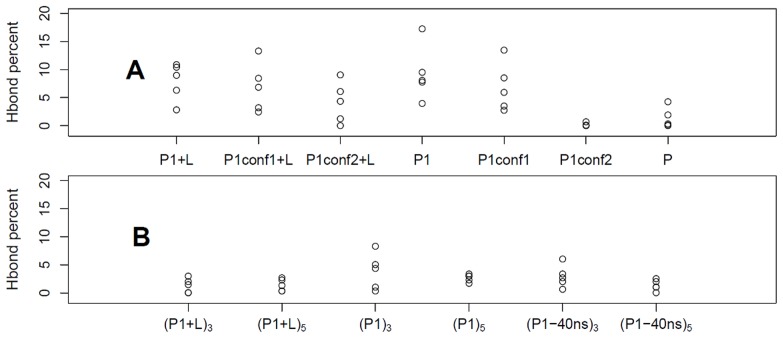
Hydrogen bond analysis. Percentages of hydrogen bond formation between LYS143 and TYR188 in each subunit along MD (A) and TAMD (B) trajectories. The hydrogen bond is considered to be formed when the minimum distance among all possible donor/acceptor distances, between the two residues, is less than 2.5 Å.

Let us discuss now the role of specific residue-residue interactions in the stabilization of the different C-loop conformations. The comparison of the X-ray crystallographic structures 2W8E (apo AChBP) and 2BYS (holo AChBP) points out that the C-loop closed conformation in the presence of lobeline is stabilized by a network of hydrogen bonds, among which is the one between LYS143 in 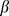 strand 7 and TYR188 in the C-loop (see Fig. S4F). In Fig. 5 we report the persistence of this hydrogen bond along our standard MD simulations, defined as the percentage of time along a full trajectory that the bond is formed. For each simulation, data are plotted for the five different protein subunits. As shown in Fig. 5A, the LYS143/TYR188 bond is very rarely formed in P, while it is more formed in most of the subunits in the presence of lobeline. Modifying the initial P1 protein structure makes the hydrogen bond percentage evolve toward the one in the native apo P state, particularly for P1conf2. These structures relaxes plausibly toward the target state, i.e. the unbound one, on the time scale of the unbiased MD simulation, pointing out where the transition bottlenecks could be located, at molecular level. Along the TAMD simulations started from the P1 and P1–40 ns initial conditions (Fig. 5B) the behavior is much more similar to the one observed in the target P protein with respect to the one along the standard MD P1. TAMD produces the evolution toward P spontaneously already at 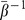 equal to 3 kcal/mol, and even more at 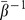 equal to 5 kcal/mol. We would like to stress that TAMD is not used here to validate the method against the simulation results on the ad hoc structures, as it could appear, but to explore the free energy underlying the C-loop transition, with the aim to overcome the known bottlenecks. A posteriori, the results obtained remark the usefulness and powerfulness of the TAMD method per se.

**Figure 6:**
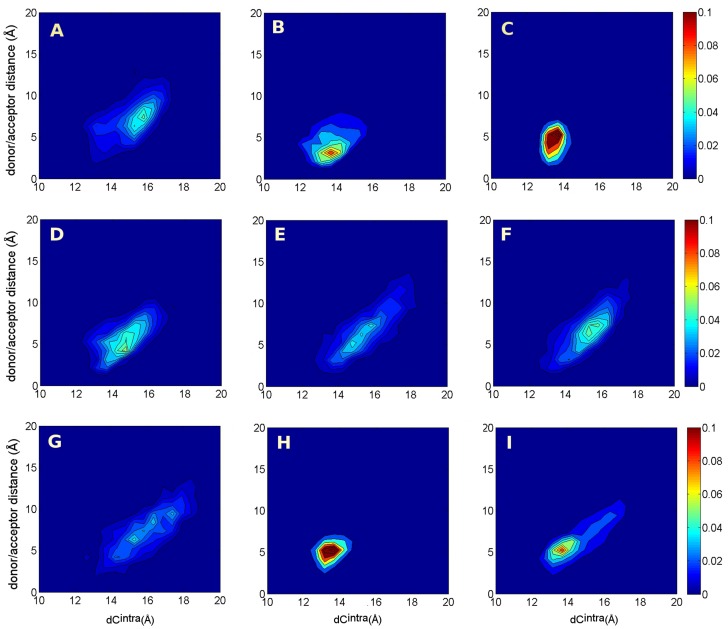
Two dimensional histograms. Two dimensional histograms n(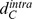, LYS143/TYR188 closest donor/acceptor distance). A: simulation P; B: P1; C: P1+L; D and E: TAMD from the P1 initial condition at 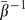 3 kcal/mol and 5 kcal/mol; F and G: TAMD from the P1-40 ns initial condition at 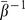 3 kcal/mol and 5 kcal/mol; H and I: TAMD from P1+L initial condition at 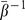 3 kcal/mol and 5 kcal/mol.

The relation between the LYS143-TYR188 hydrogen bond and the degree of C-loop closure is better evidenced by plotting the two dimensional histograms of 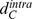 and the distance between LYS143 and TYR188 donor/acceptor atoms. In Fig. 6 we show such maps calculated over all unbiased MD trajectories (panels A–C). When the bond is formed or partially formed, as in P1 and P1+L (Fig. 6, panels B and C), the C-loop is in the closed state; in correspondence to larger LYS143-TYR188 distances as in P (panel A), the C-loop is mainly in the open form.

The strength of the LYS143-TYR188 bond and the possibility that its formation may contribute to the C-loop closure was already investigated for the apo AChBP in [50], where the potential of mean force for the interaction of the two above mentioned residues was calculated using umbrella sampling on the LYS143NZ-TYR188OH distance. In that work, the bound-like conformation was stabilized by about 2.0 kcal/mol at a distance of 3Å. The strength of the hydrogen bond was found somewhat weak; according to the authors, this could arise from the flexibility of the C-loop and/or concurrent interactions of both residues with water molecules.

At variance with monodimensional PMF calculation, we study the transition in the full protein, i.e. we accelerate all C-loops together. According to our results, the formation and persistence in time of the bond between LYS143 and TYR188 is strictly anti-correlated to the presence of the lobeline. The hydrogen bond is weak and transiently formed in P1+L, as distance values larger than 3Å are observed (see Fig. 6, panel C), while the distribution in P1 show a larger population in the range 2–4 Å (Fig. 6, panel B). The weakness in P1+L is brought about by the transient hydrogen bond that the lobeline could establish with TYR188 (see below). Therefore, in AChBP bound to lobeline, the hydrogen bond between LYS143 and TYR188 does not have a dominant structural role in maintaining the C-loop closed.

The relation between the LYS143/TYR188 donor/acceptor distance and 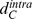 was followed also along the TAMD trajectories. Results are shown in Fig. 6, for the TAMD starting from P1 and P1-40ns at 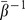 equal to 3 and 5 kcal/mol (panels D to G), confirming i) how TAMD was able to efficiently push the P1 protein structure out of the basin in which it was trapped along the 150ns MD trajectory (see Fig. 6, panel B), already at 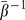 equal to 3 kcal/mol (see Fig. 6, panel D); ii) the higher energy cost for opening the C-loops and disrupting the LYS143/TYR188 bond when the lobeline is present in the binding pocket, as pointed out by the comparison of the maps in panels H, I.

Another hydrogen bond that has been long discussed for AChBP is the one between SER146 and TYR93 (see Fig. S4A). It has been suggested that TYR93 has a role as “gatekeeper” in AChBP [77]: depending on its position, it makes the lobeline pocket inaccessible (as in 2W8E) or open (as in 2BYS). Furthermore, experimental results on full nicotinic receptor [78] suggest that mutating TYR93 (in particular 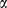
Y93W) causes a change in the structure of the binding pocket that compromises both the mobility of the ligand into and out of its docking site, and the speed of channel opening.

**Figure 7:**
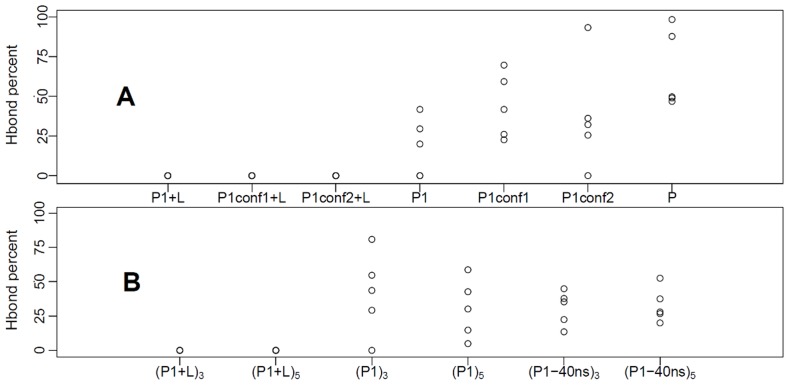
Hydrogen bond analysis. Percentages of hydrogen bond formation between SER146 and TYR93 in each subunit along MD (A) and TAMD (B) trajectories. The hydrogen bond is considered to be formed when the minimum distance among all possible donor/acceptor distances, between the two residues, is less than 2.5Å.

In Fig. 7 we report the persistence in time, along our simulations, of the SER146/TYR93 bond: in P the bond is always present (Fig. 7A), while in P1+L it is never formed; this is due to the presence of the lobeline in the binding pocket. As it will be shown below, the carbonyl group of SER146 interacts with the lobeline hydroxyl group along the entire P1+L trajectory, in four out of five subunits. In the unbiased P1 trajectory, an intermediate behavior between P and P1+L is observed. Once again, biasing the initial P1 protein structure makes the hydrogen bond percentage to evolve toward the one in the natural P conformation, particularly for P1conf2.

**Figure 8:**
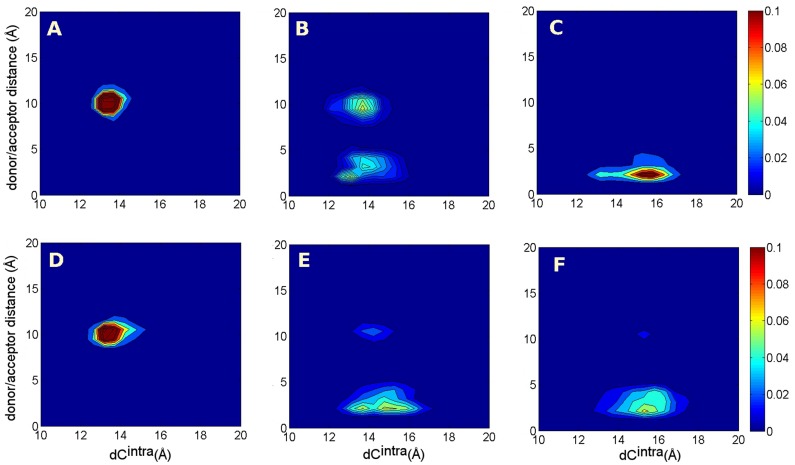
Two dimensional histograms. Two dimensional histograms n(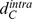, SER146/TYR93 closest donor/acceptor distance). A: MD simulation P1+L; B: P1; C: P; D: TAMD simulation (P1+L)_3_; E: (P1)_3_; F: (P1-40ns)_3_.

Along the TAMD simulations started from the P1 and P1-40ns initial conditions (Fig. 7B) the persistence in time becomes much more similar to the one observed in the target P protein with respect to the one along the standard MD P1. TAMD produces the evolution toward P spontaneously already at 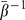 equal to 3 kcal/mol. In analogy with the LYS143/TYR188 bond, the two dimensional histograms shown in Fig. 8 point out a relation between close/open C-loops and the formation/disruption of the SER146/TYR93 bond. It is clearly shown how TAMD was efficient in driving the system toward the natural P state already at 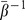 3 kcal/mol; this was better achieved by starting from the P1-40ns initial condition.

The features of the SER146/TYR93 bond in the TAMD exploration are further discussed in Text S1 (see section “ TAMD exploration and the role of SER146/TYR93”, Fig. S8, Fig. S9 and Fig. S10).

### AChBP-lobeline Interaction

**Figure 9:**
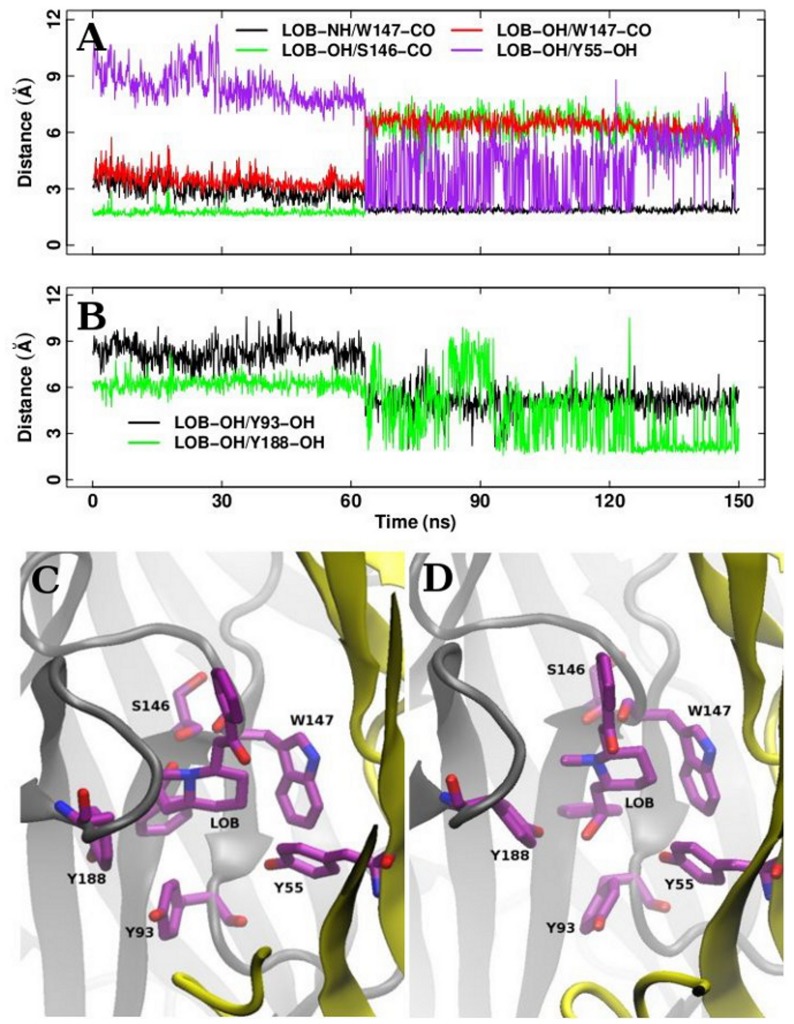
The ligand transition. A and B: residues-lobeline donor/acceptor distances (see legends); C: lobeline and protein residues configuration before the OH transition; D: after the OH transition.

We now turn to discuss the lobeline/AChBP interactions and conformations along our MD and TAMD simulations of the complex systems. At first, we monitored [27]: (i) four hydrogen bonds connecting lobeline carbonyl and the hydrogen atom H
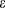
1 of TRP147, lobeline amide and carbonyl of TRP147, lobeline hydroxyl and carbonyl of SER146, and lobeline hydroxyl and carbonyl of TRP147; (ii) three van der Waals interactions: between lobeline piperidine ring and TRP147 indole, between lobeline methyl and TYR188 aromatic ring, and between lobeline methyl and TYR195 aromatic ring. The hydrogen bonds are monitored through the distances between hydrogen donor and acceptor atoms. The van der Waals interactions are monitored through the distances between the centers of mass of the two atom groups. Among the hydrogen bonds observed in the crystallographic structure 2BYS [27], only one, between the lobeline hydroxyl group (LOB-OH) and the SER146 carbonyl (SER146-CO) is formed during the MD simulations (Table S1). Between the rings of TYR188 and TYR195, the lobeline methyl (LOB-CH3) seems to prefer to interact with TYR195 ring, as the distances LOB-CH3/TYR195-ring are smaller than the ones observed for LOB-CH3/TYR188-ring (Table S1). This marked difference from the X-ray crystallographic structure can be explained by the fact that only the global electronic envelop of the ligand was visible in the Xray crystallographic structure 2BYS, thus hampering a precise definition of the ligand conformation. Among the protein-lobeline distances monitored on the trajectory P1+L, in the subunit P3, the distance between the lobeline hydroxyl (LOB-OH) and the carbonyl groups of SER146 and TRP147, displays a strong transition around 64 ns (Fig. 9A, green and red curve, respectively). This transition is accompanied by the formation of a hydrogen bond between the lobeline amide (LOB-NH) and the carbonyl group of TRP147 (Fig. 9A, black curve). An examination of frames extracted in the 65–100 ns range of P1+L reveals that one of the aromatic rings of lobeline changed its rotameric state around 64 ns, and the LOB-OH then pointed toward the complementary subunit, as represented in Fig. 9D, establishing transient hydrogen bonds with the hydroxyls of the TYR55 (Fig. 9A, purple curve), TYR93 (Fig. 9B, black curve) and TYR188 (Fig. 9B, green curve). This movement of lobeline is an isolated event, which might describe the initial steps of lobeline dissociation.

The ligand lobeline has been shown to have a partial agonist effect [79], on 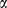
7 [8] and 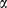
4
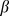
2 [9] nAChRs. Although it is not clear what partial agonism means at the molecular level [80], the observation of the lobeline transition would support a model where a partial agonist is an agonist ligand that interacts weakly with the receptor and is thus more prone to dissociation. On the other hand, it has been observed that other partial agonists co-crystallized with AChBP cause an incomplete closure of loop C against the binding site [32]; at variance, in 2BYS the C-loop is capped around the lobeline, similar to what observed with full agonists. This has been also observed in the recent Xray crystallographic structure of AChBP from *Capitella teleta*, bound to lobeline [81]. Our results (see the 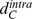 histogram in P1+L in Fig. 3) show that the lobeline-induced tight closing is kept during the simulations. The lobeline OH transition would reconcile these two points of view as the lobeline weakened interaction would arise from isolated dissociation events rather than from different, induced, conformations of C-loop.

In the biased MD simulations P1conf1+L and P1conf2+L, the lobeline transition was not observed. This could be explained by considering that the starting points were produced by modifying, among others, the orientation of GLN186, which strongly influences the position of TYR188 (data not shown).

**Figure 10:**
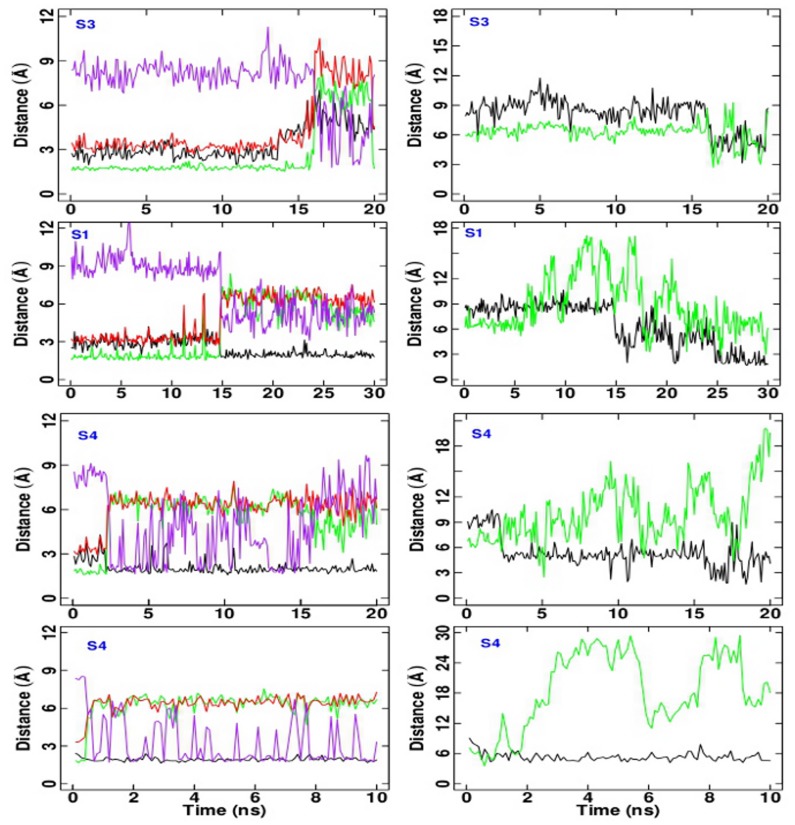
Protein-lobeline donor/acceptor distances. Protein-lobeline donor/acceptor distances involved in the ligand transition in some representative subunits along the TAMD trajectories. First row from top:(P1+L)_3_ trajectory; second row: (P1+L)_5_; third row: (P1+L)_7_; fourth row: (P1+L)_10_. Left column: LOB-NH/W147-CO (black), LOB-OH/S146-CO (green), LOB-OH/W147-CO (red) and LOB-OH/Y55-OH (purple) hydrogen bond distances. Right column: LOB-OH/Y93-OH (black) and LOB-OH/Y188-OH (green) hydrogen bond distances.

The lobeline transition is however observed in TAMD trajectories (Table S2), and significantly accelerated in at least one subunit (see Fig. 10 for a representative subunit in each TAMD trajectory). In particular, it is observed progressively in more than one subunit as the effective temperature increases. At 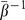 equal to 3 kcal/mol it occurs in one subunit only, namely P3 at about 15 ns. At 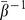 5 kcal/mol it occurs in three out of five subunits: P1 first at about 15 ns, then P4 at about 22.5 ns and finally in P5 about 27 ns. At 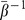 equal to 7 kcal/mol, it occurs in four out of five subunits: P4 first at 2.5 ns, then P3 at about 5 ns, followed by P5 at about 10 ns and finally in P1 at about 18 ns. At the highest 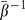, 10 kcal/mol, the event is observed in the five subunits at the following time frames along the trajectory: P4 at about 0.6 ns, P3 at about 3 ns, P2 at about 3.3 ns, P5 at about 6.4 ns, and P1 at about 6.6 ns. Remarkably, we observe that the same chain of events characterizes the lobeline transition in MD and TAMD, i.e. we observed the same key residues bonds rupture/formation events (see Figs. 9 and 10).

**Figure 11:**
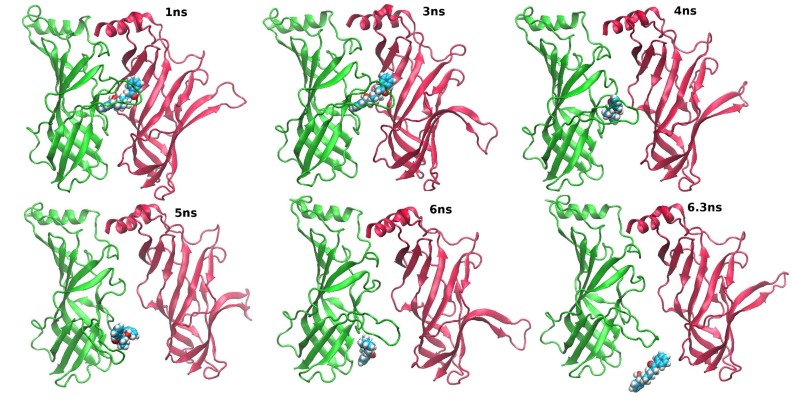
Snapshots along the TAMD trajectory. Snapshots taken at 1(P1+L)_10_ trajectory for S3–S2 interface. S3 is in green; S2 is in red, cartoon representation; lobeline in VDW representation. Lobeline is fully in the solvent at 6.3 ns.

In the TAMD at 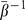 equal to 10 kcal/mol, the dissociation of the lobeline from the binding pocket is complete after few nanoseconds (Fig. 11). The ligand exits from the binding site and migrates downward to the solvent; its motion is assisted by a corresponding downward motion of the C-loop. A similar path was obtained for the unbinding of acetylcholine from the ECD of 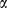
7 nicotinic receptor using Steered MD in [57]. In that work, the free energy difference between the bound and unbound states was estimated to be about 10–15 kcal/mol, consistent with the effective energy adopted by us. At variance with the paths proposed by Zhang et al. [57] however, the dissociation path observed in the present work was not obtained by forced pulling but occurred spontaneously in the TAMD trajectory. A downward motion like the one we observed would be not supported by the analysis of several crystal structures of AChBP both in the holo and the apo state, in which it is shown that the loop C assumes different intermediate positions mainly in the direction outwards perpendicular to the main axis of the protein. We would like to stress that the ligand dissociation trajectory observed here is a possible one as it is provided by TAMD. Indeed, TAMD trajectories are likely to pass close the most probable reaction path [69] although there is no explicit requirement for this. In particular, the first step of lobeline dissociation is additionally supported as the most plausible part of the dissociation because the same event is spontaneously observed along the MD simulation of the native lobeline-bound complex.

## Discussion

In the present work, the transition of the *Aplysia californica* AChBP from the holo to apo state was explored using MD and TAMD simulations. The transition has been qualitatively described using: (i) opening distances of C-loop, (ii) individual hydrogen bonds. The TAMD simulations have been shown to dramatically accelerate the transition observed in some MD simulations only after some ad hoc modifications of the structure. Indeed, to get the holo/apo transition of the loop C in unbiased MD simulations it was needed to modify the native structure of the apo protein (P1), by disrupting the pattern of some key hydrogen bonds. Possible first steps of spontaneous lobeline dissociation by TAMD have been observed. At the highest effective temperature, a path of complete ligand dissociation has been observed in one subunit.

The analysis of MD simulations has shown that the AChBP states bound to lobeline and apo are stable within an interval of 150 ns. This agrees with the timescale of channel opening in nAChRs, which is on the order of 1 ms [49]. AChBP bound to lobeline shows along the MD trajectories limited oscillations around a well defined mean structure. On the contrary, the apo AChBP obtained from 2W8E has a much less defined conformational state due to the large C-loop internal mobility, although nAChRs in the corresponding apo state display a well-defined physiological state with a closed channel.

The initial change of orientation of few residues (ARG57, ARG95, GLN103, GLN184, LYS143) is sufficient to reduce, at least in part, the long metastability of AChBP in standard MD trajectories. These residues have been selected as they display a variability of orientations in the AChBP crystal 2BYS. This variability in the crystal is probably the sign of an equilibrium of AChBP in solution among several conformations. It is interesting to see that pushing the conformations of these mobile residues toward the ones observed in the absence of lobeline (PDB entry: 2W8E), induce a whole transition of AChBP toward the apo state, as seen by monitoring the C-loop behavior. Thus, the relevance of these residues modifications is supported by the plausible effects the modifications have on the ACHBP. One should notice that, among the modified residues, only GLN184 is located in C-loop, and only LYS143 directly interacts with C-loop. Most of the residues are thus located apart from the region displaying the major drift during the transition. This is the sign of a domino effect relating the conformation of these residues to the C-loop opening [82].

The TAMD simulation produces spontaneously the holo-to-apo transition, accelerating by a factor of 10 the transition obtained as described above by altering the initial configuration in standard MD. The TAMD results give very qualitative estimation of the energy barriers which are crossed during the transition, through the observation of the effective energy 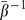 values for which the conformational transition is observed. The value is in the range 3–5 kcal/mol for the AChBP transition, which is found to be in qualitative agreement with the free energy barrier of about 2 kcal/mol between the open and closed C-loop states of the ECD of 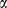
7 nAChR, calculated by umbrella sampling using the LYS143/TYR188 bond as collective variable [50]. In the presence of lobeline, the acceleration of the transition by TAMD is reduced, and the transition, not seen at 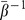 = 3 kcal/mol, starts to be observed from 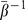 equal to 5 kcal/mol. The increase of about 2 kcal/mol induced by the presence of lobeline is in qualitative agreement with the affinity of 0.3 nM [27] experimentally measured for this ligand on *Aplysia californica* AChBP. Interestingly, the TAMD acceleration also influences the early steps of lobeline dissociation. Indeed, the complete dissociation of lobeline from the binding pocket is achieved, for 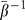 equal to 10 kcal/mol, in the nanosecond timescale.

The analysis of the hydrogen bonds along standard MD and the TAMD trajectories permitted to describe the transition from the holo to the apo state in terms of the evolution of a network of interactions. Among the hydrogen bonds, the one between LYS143 and TRP188 has been shown previously in the literature [50] to be important. But, also other hydrogen bonds like SER146/TYR93 have been found even more relevant to the mechanism of the transition, as pointed out by the TAMD simulations. Most of the residues involved in hydrogen bonds are conserved in nAChRs and we can thus expect similar behavior for them in nAChRs. Their importance on the binding/gating events in nAChRs has been already tested by site direct mutagenesis [83], [84], validating the model proposed here.

All the observed conformational changes are quite local, but nevertheless relevant according to what it has proposed in the literature, in particular by Auerbach and coworkers, in the so-called conformational wave model [85], [86] where the ensemble of variations in local interactions is thought to produce the overall allosteric mechanism. The results obtained here on AChBP could be then fully relevant for the nAChRs. Furthermore, the effects of lobeline on the TAMD transition agrees with the lobeline function on nAChRs as full agonist, as the presence of lobeline decrease or suppress the acceleration of the C-loop opening.

## Materials and Methods

### Starting Structures

It is known that Acetylcholine (ACh) is the natural ligand of AChBP, but, up to now, there is no X-ray crystallographic structure of AChBP bound to ACh. As it is quite important to start from a ligand position assessed by precise information from structural biology, a high-resolution structure of AChBP bound to lobeline was used, rather than a model obtained by docking ACh to an empty AChBP structure. Moreover, the choice of lobeline as ligand is based on its therapeutic importance [8], [9]. As starting conditions for the apo and holo AChBP conformation we used the X-ray crystallographic structures PDB entry names: 2W8E [33] and 2BYS [27] of *Aplysia californica* AChBP. They were the highest resolution AChBP structures available (2.05 Å for 2BYS and 1.9 Å for 2W8E), at the time this work started (a new AChBP apo structure has been deposited later, PDB entry: 2Y7Y at 1.89 Å). Missing residues in structures 2BYS (first loop after the helix 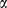
1) and 2W8E (in loop C) were reconstructed using homology modeling with Modeller v9.8 [87], [88]. In what follows, we refer to the original residue numbering in 2BYS [27]. Note that residue numbering is shifted by +2 with respect to the original numbering in 2W8E [33].

In order to investigate the role of selected residues on the stability of the AChBP conformations, we modified their arrangement in the initial protein conformations thus creating alternative starting points for the lobeline-bound AChBP structure. Details are fully given in Text S1 (see Section “New AChBP models”).

### MD and TAMD Simulations

MD simulations of AChBP were carried out for several systems in explicit water: the apo pentamer (P), the lobeline-bound pentamer in the presence (P1+L) or in the absence (P1) of lobeline. The starting points for P and P1+L/P1 simulations were respectively the first five chains, called A to E, of the PDB entries 2W8E and 2BYS, completed as explained. Starting from the new AChBP structures, additional MD simulations were carried out both in the presence and in the absence of lobeline. Set up of the systems and the standard MD simulation protocol are fully described in Text S1 (see section “Molecular Dynamics simulations”).

As for TAMD, the theoretical basis was originally presented by Maragliano and Vanden-Eijnden [58]. The coupled system of equations describing TAMD, for a system whose configuration is specified by 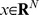, is:
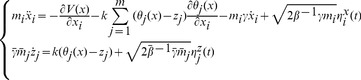
(1)where 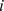 = 1,…3N and 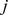 = 1,…m. 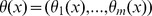 are collective variables (CVs) that are functions of the atomic Cartesian coordinates and 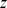 = (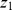,…,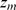) is a set of particular values of these variables; 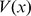 is the inter-atomic MD potential; 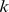 is the spring constant coupling the CVs with their target values; 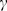 and 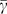 are friction coefficient; 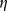 is a white-noise process, i.e. a Gaussian process with mean zero and covariance 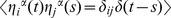 where 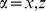; 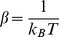 where 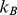 is the Boltzmann constant and the 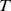 is the system temperature; 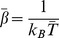 where 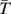 is an artificial temperature, 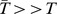. The aforementioned set of equations describe the motion of 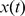 and 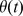 over the following extended potential:



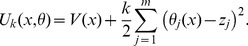
(2)The advantage of TAMD [58], is that if (i) 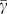 is chosen sufficiently large so as to guarantee that 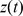 evolves much more slowly relative to the fundamental variables, and (ii) 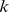 is sufficiently large such that 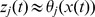 at any given time, then the z variables effectively evolve according to the equation:
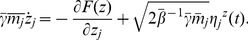
(3)where 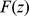 is the free energy associated to the CVs 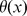. Therefore the points in the CVs space are sampled according to the Boltzmann factor 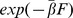 which is tuned to be more uniform than the associated physical Boltzmann factor 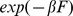 by taking 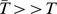. TAMD therefore accelerates the sampling of the conformational landscape through the CVs space by increasing the probability of visiting points with relatively low physical Boltzmann factors.

The procedure for determining suitable values of 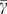 and spring constant 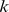 is described in Text S1 (see Section “Determining the value of fictitious friction 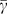 and spring constant 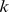
”, and Fig. S6). TAMD trajectories were initiated from: (i) the 40 ns frame of MD trajectories P1+L and P1, (ii) the 40 ns frame of the P1+L trajectory, to which the lobeline has been removed.

As collective variables we used the Cartesian coordinates of the center of mass of the loop C (residues 183–194) and cys-loop (residues 128–140) of each subunit of AChBP, for a total of 30 variables. We could do this because, when compared with other available enhanced sampling methods, TAMD allows the use of a larger number of variables. Since we used a large number of collective variables and we observed the opening mechanism in the TAMD simulations, we did not compare alternative choices. A posteriori, the analysis of two different distances to assess C-loop opening, including the distance between the loop and the facing monomer surface (see below, “Trajectory analysis”), shows that although these distances are not directly evolved in TAMD, they sample the range of values from the closed to the open state. In particular, we have considered “open” those conformations that showed a value of the distance similar to the one in the crystal structure 2W8E.

TAMD simulations were run from 10 up to 30 ns, with temperature 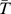 of about 1500 K, 2500 K, 3500 K and 5000 K, corresponding to 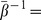 3, 5, 7 and 10 kcal/mol. In TAMD runs, the atomic force coming from the coupling potential and the evolution of the CVs are implemented via a Tcl script linked to the NAMD code.

A list of the MD simulations started from the original conformations and of the TAMD simulations is given in Table 1, together with other MD simulations started from modified conformations. Systems set-up details are given in Table S3.

### Trajectory Analysis

In order to characterize the protein conformational changes, we used, following [89], three distances between the centers of mass of C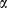 atom groups. For the loop C, the distance 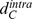 (Fig. S5A) internal to each subunit, is calculated between the center of mass of residues 183–194 of loop C in the principal subunit and the center of mass of the residues 143 and 144. For the cys-loop, two distances 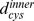 (Fig. S5B) and 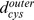 (Fig. S5C) are defined, between the cys-loop center of mass (residues 128–140) and the center of mass of residues 179–181,198–204 and 120–127,49–60,30–43, found respectively in the inner and outer 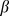-sheet of the same subunit. These 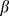-sheet residues were picked up as the ones displaying average Mean Square Fluctuations smaller than 0.8 Å in the P simulation (see Results section), to guarantee a “rigid” back-wall against which the cys-loop displaces. To study the role of specific residue-residue interactions, an intra-protein hydrogen bond analysis has been performed along all MD trajectories (see Fig. 5, Fig. 7 and Fig. S7). Details are fully described in Text S1, see Section on “Hydrogen bond analysis”.

## Supporting Information

Figure S1 **Structure of lobeline.** Schematic structure of the lobeline molecule.
(TIFF)

Figure S2
**Root Mean Square Deviation of individual subunits.** Root Mean Square Deviation (in Å) of individual subunits calculated from the starting conformations (after equilibration) as 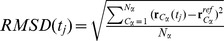, where 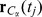 is the position of the 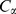 atom at 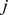
th time step, 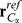 is the position of the 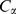 atom in the reference structure, 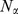 is the total number of 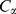 atoms in the subunit. Upper panel: P; central panel: P1; lower panel: P1+L. The RMSD values are calculated after removing the roto-translational body motions of the single subunits [90]. The curves are colored according to the scheme in Fig. 1 in Main text.(TIFF)

Figure S3
**Distribution of 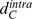 values.** Distribution of 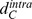 values (Å) calculated over the TAMD trajectory at different time slices (see legends), at 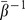 = 10 kcal/mol. Upper panel: (P1)_10_; lower panel: (P1+L)_10_.(TIFF)

Figure S4 **Protein residues involved in hydrogen bonds.** Protein residues (in licorice) involved in hydrogen bonds analysed in this work. The protein is shown as a cartoon model. In panels C and D, the principal and complementary subunits are shown in gray and yellow, respectively.
(TIFF)

Figure S5
**Schematic representation of the C-loop and cys-loop distances.** Schematic representing of the C-loop and cys-loop distance parameters analyzed in this work. In A) the blue line indicates the 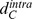; B) 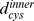; C) 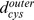. Protein is shown as a cartoon model.(TIFF)

Figure S6
**Running average of the restraining force for each CV and 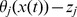 versus time.** A) Running average of the restraining force for each CV, during MD simulation in which the CVs are fixed to their initial values; 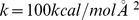. B) 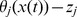 values versus time for restrained dynamics, 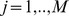 where 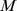 is the total number of collective variables; 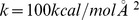.(TIFF)

Figure S7
**Hydrogen bond analysis.** Number of subunits for which hydrogen bonds are present 50–90% of the time (A,C) or more than 90% of the time (B,D). The analyzed trajectories are MD (A,B) and TAMD at 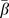 = 3 or 5 kcal/mol (C,D). The residue pairs are: ASP159/ARG59 (black), GLU153/ARG79 (cyan), GLU193/ARG79 (magenta), GLU193/LYS25 (green). The hydrogen bond is considered to be formed when the minimum distance among all possible donor/acceptor distances, for each pair, is less than 2.5 Å.(TIFF)

Figure S8
**Probability of all atoms SER146 and TYR93 RMSD.** Probability of all atoms SER146 and TYR93 RMSD value less than 2 Å from the reference states: P/1, P/2, P1+L, P/1, & P/2, P/1 & P/2 & P1+L, P/1 & P1+L, P/2 & P1+L, along the TAMD trajectories P1 (black bar) and P1-40ns (dark gray bar) at 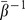 = 3 kcal/mol.(TIFF)

Figure S9 **Probability of all atoms SER146 and TYR93 RMSD.** As in Fig. 19, along the TAMD trajectories (P1)_5_ (black bar); (P1)_7_ (dark gray bar) and (P1)_10_ (gray bar).
(TIFF)

Figure S10 **Probability of all atoms SER146 and TYR93 RMSD.** As in Fig. 19, along the TAMD trajectories (P1+L)_3_ (black bar), (P1+L)_5_ (dark gray bar), (P1+L)_7_ (gray bar) and (P1+L)_10_ (light gray bar).
(TIFF)

Table S1 **Distances between lobeline and AChBP atoms.**
(PDF)

Table S2 **Distances between lobeline and AChBP atoms.**
(PDF)

Table S3 **Size and contents of the simulated systems.**
(PDF)

Text S1 (PDF)

## References

[1] Karlin A (2002) Emerging structure of the nicotinic acetylcholine receptors. Nat Rev Neurosci 3: 102114. 10.1038/nrn731 11836518

[2] Lester HA, Dibas MI, Dahan DS, Leite JF, Dougherty DA (2004) Cys-loop receptors: new twists and turns. Trends Neurosci 27: 329336. 10.1016/j.tins.2004.04.002 15165737

[3] Changeux JP, Edelstein SJ (2005) Nicotinic Acetylcholine Receptors: From Molecular Biology to Cognition. 1st ed, Odile Jacob, New York.

[4] Changeux JP, Edelstein SJ (2005) Allosteric mechanisms of signal transduction. Science 308: 1424–1428. 15933191 10.1126/science.1108595

[5] Taly A, Corringer PJ, Guedin D, Lestage P, Changeux JP (2009) Nicotinic receptors: allosteric transitions and therapeutic targets in the nervous system. Nat Rev Drug Discov 8: 733–750. 19721446 10.1038/nrd2927

[6] Whatley VJ, Harris RA (1996) The cytoscheleton and neurotrasmitter receptors. Int Rev Neurobiol 39: 113–143. 8894846 10.1016/s0074-7742(08)60665-0

[7] Larsson A, Engel JA (2004) Neurochemical and behavioral studies on ethanol and nicotine interactions. Neurosci Biobehav Rev 27: 713–720. 15019421 10.1016/j.neubiorev.2003.11.010

[8] Briggs CA, McKenna DG (1998) Activation and inhibition of the human alpha7 nicotinic acetylcholine receptor by agonists. Neuropharmacology 37: 1095–1102. 9833639 10.1016/s0028-3908(98)00110-5

[9] Miller DK, Crooks PA, Dwoskin LP (2000) Lobeline inhibits nicotine-evoked [(3)H]dopamine overflow from rat striatal slices and nicotine-evoked (86)Rb(+) efflux from thalamic synaptosomes. Neuropharmacology 39: 2654–2662. 11044735 10.1016/s0028-3908(00)00140-4

[10] Unwin N (2005) Refined structure of the nicotinic acetylcholine receptor at 4 Å resolution. J Mol Biol 346: 967–989. 15701510 10.1016/j.jmb.2004.12.031

[11] Unwin N, Fujiyoshi Y (2012) Gating movement of acetylcholine receptor caught by plunge freezing. J Mol Biol 422: 617–634. 22841691 10.1016/j.jmb.2012.07.010 PMC3443390

[12] Nury H, Renterghem CV, Weng Y, Tran A, Baaden M, et al (2011) X-ray structures of general anaesthetics bound to a pentameric ligand-gated ion channel. Nature 469: 428–431. 21248852 10.1038/nature09647

[13] Hilf RJ, Bertozzi C, Zimmermann I, Reiter A, Trauner D, et al (2010) Structural basis of open channel block in a prokaryotic pentameric ligand-gated ion channel. Nat Struct Mol Biol 17: 1330–1336. 21037567 10.1038/nsmb.1933

[14] Nury H, Poitevin F, Renterghem CV, Changeux JP, Corringer PJ, et al (2010) One-microsecond molecular dynamics simulation of channel gating in a nicotinic receptor homologue. Proc Natl Acad Sci USA 107: 6275–6280. 20308576 10.1073/pnas.1001832107 PMC2852019

[15] Hilf RJ, Dutzler R (2009) Structure of a potentially open state of a proton-activated pentameric ligand-gated ion channel. Nature 457: 115–118. 18987630 10.1038/nature07461

[16] Bocquet N, Nury H, Baaden M, Poupon CL, Changeux JP, et al (2009) X-ray structure of a pentameric ligand-gated ion channel in an apparently open conformation. Nature 457: 111–114. 18987633 10.1038/nature07462

[17] Zimmermann I, Dutzler R (2011) Ligand activation of the prokaryotic pentameric ligand-gated ion channel ELIC. PLoS Biol 9: e1001101. 21713033 10.1371/journal.pbio.1001101 PMC3119659

[18] Hilf RJ, Dutzler R (2008) X-ray structure of a prokaryotic pentameric ligand-gated ion channel. Nature 452: 375–379. 18322461 10.1038/nature06717

[19] Pan J, Chen Q, Willenbring D, Yoshida K, Tillman T, et al (2012) Structure of the Pentameric Ligand-Gated Ion Channel ELIC Co-Crystallized with its Competitive Antagonist Acetylcholine. Nat Commun 3: 714. 22395605 10.1038/ncomms1703 PMC3316889

[20] Brannigan G, Bard DNL, Henin J, Eckenhoff RG, Klein M (2010) Multiple binding sites for the general anesthetic isoflurane indentified in the nicotinic acetylcholine receptor trasmembrane domain. Proc Nat Acad Sc 107: 14122–14127. 20660787 10.1073/pnas.1008534107 PMC2922517

[21] Pan J, Chen Q, Willenbring D, Mowrey D, Kong XP, et al (2012) Structure of the pentameric ligand-gated ion channel GLIC bound with anesthetic ketamine. Structure 20: 1463–1469. 22958642 10.1016/j.str.2012.08.009 PMC3446250

[22] Prevost MS, Sauguet L, Nury H, Renterghem CV, Huon C, et al (2012) A locally closed conformation of a bacterial pentameric proton-gated ion channel. Nat Struct Mol Biol 19: 642–649. 22580559 10.1038/nsmb.2307

[23] Spurny R, Billen B, Howard RJ, Brams M, Debaveye S, et al (2013) Multisite binding of a general anesthetic to the prokaryotic pentameric Erwinia chrysanthemi ligand-gated ion channel (ELIC). J Biol Chem 288: 8355–8364. 23364792 10.1074/jbc.M112.424507 PMC3605653

[24] Sixma K, Smit AB (2003) Acetylcholine binding protein (AChBP): a secreted glial protein that provides a high-resolution model for the extracellular domain of pentameric ligand-gated ion channels. Annu Rev Biophys Biomol Struct 32: 311–334. 12695308 10.1146/annurev.biophys.32.110601.142536

[25] Taly A, Colas C, Malliavin T, Blondel A, Nilges M, et al (2011) Discrimination of agonists versus antagonists of nicotinic ligands based on docking onto AChBP structures. J Mol Graph Model 30: 100–109. 21764343 10.1016/j.jmgm.2011.06.008

[26] Brejc K, van Dijk WJ, Klaassen RV, Schuurmans M, van Der Oost J, et al (2001) Crystal structure of an ACh-binding protein reveals the ligand-binding domain of nicotinic receptors. Nature 411: 269–276. 11357122 10.1038/35077011

[27] Hansen SB, Sulzenbacher G, Huxford T, Marchot P, Taylor P, et al (2005) Structures of Aplysia AChBP Complexes with Nicotinic Agonists and Antagonists Reveal Distinctive Binding Interfaces and Conformations. EMBO J 24: 2625–3646. 10.1038/sj.emboj.7600828 PMC1276711 16193063

[28] Celie PH, van Rossum-Fikkert SE, van Dijk WJ, Brejc K, Smit AB, et al (2004) Nicotine and carbamylcholine binding to nicotinic acetylcholine receptors as studied in AChBP crystal structures. Neuron 41: 907–914. 15046723 10.1016/s0896-6273(04)00115-1

[29] Celie PH, Kasheverov IE, Mordvintsev DY, Hogg RC, van Nierop P, et al (2005) Crystal structure of nicotinic acetylcholine receptor homolog AChBP in complex with an alpha-conotoxin PnIA variant. Nat Struct Biol 12: 582–588. 10.1038/nsmb951 15951818

[30] Celie PH, Klaassen RV, van Rossum-Fikkert SE, van Elk R, van Nierop P, et al (2005) Crystal structure of acetylcholine-binding protein from Bulinus truncatus reveals the conserved structural scaffold and sites of variation in nicotinic acetylcholine receptors. J Biol Chem 280: 26457–26466. 15899893 10.1074/jbc.M414476200

[31] Hibbs RE, Radic Z, Taylor P, Johnson DA (2006) Influence of agonists and antagonists on the segmental motion of residues near the agonist binding pocket of the acetylcholine-binding protein. J Biol Chem 281: 39708–39718. 17068341 10.1074/jbc.M604752200

[32] Hibbs RE, Sulzenbacher G, Shi J, Talley TT, Conrod S, et al (2009) Structural determinants for interaction of partial agonists with acetylcholine binding protein and neuronal alpha7 nicotinic acetylcholine receptor. EMBO J 28: 3040–3051. 19696737 10.1038/emboj.2009.227 PMC2760105

[33] Ulens C, Akdemir A, Jongejan A, van Elk R, Bertrand S, et al (2009) Use of acetylcholine binding protein in the search for novel alpha7 nicotinic receptor ligands, *In silico* docking, pharmacological screening, and X-ray analysis. J Med Chem 52: 2372–2383. 19331415 10.1021/jm801400g

[34] Ulens C, Hogg RC, Celie PH, Bertrand D, Tsetlin V, et al (2006) Structural determinants of selective alpha-conotoxin binding to a nicotinic acetylcholine receptor homolog AChBP. Proc Natl Acad Sci U S A 103: 3615–3620. 16505382 10.1073/pnas.0507889103 PMC1450132

[35] Bourne Y, Hansen SB, Sulzenbacher G, Talley TT, Huxford T, et al (2006) Structural comparison of three crystalline complexes of a peptidic toxin with a synaptic acetylcholine recognition protein. J Mol Neurosci 30: 103–104. 17192648 10.1385/JMN:30:1:103 PMC4755294

[36] Bourne Y, Radic Z, Aráoz R, Talley TT, Benoit E, et al (2010) Structural determinants in phycotoxins and AChBP conferring high affinity binding and nicotinic AChR antagonism. Proc Natl Acad Sci USA 107: 6076–6081. 20224036 10.1073/pnas.0912372107 PMC2851920

[37] Bourne Y, Talley TT, Hansen SB, Taylor P, Marchot P (2005) Crystal structure of a CBTX-AChBP complex reveals essential interactions between snake alpha-neurotoxins and nicotinic receptors. EMBO J 24: 112–115. 10.1038/sj.emboj.7600620 PMC1142565 15791209

[38] Hansen SB, Taylor P (2007) Galanthamine and non-competitive inhibitor binding to ACh-binding protein: evidence for a binding site on non-alpha-subunit interfaces of heteromeric neuronal nicotinic receptors. J Mol Biol 369: 895–890. 17481657 10.1016/j.jmb.2007.03.067 PMC2031909

[39] Dutertre S, Ulens C, Buttner R, Fish A, van Elk R, et al (2007) AChBP-targeted alpha-conotoxin correlates distinct binding orientations with nAChR subtype selectivity. EMBO J 26: 3858–3860. 17660751 10.1038/sj.emboj.7601785 PMC1952216

[40] Edink E, Rucktooa P, Retra K, Akdemir A, Nahar T, et al (2011) Fragment Growing Induces Conformational Changes in Acetylcholine-Binding Protein: A Structural and Thermodynamic Analysis. J Am Chem Soc 133: 5363–5371. 21322593 10.1021/ja110571r

[41] ShahsavarA, KastrupJS, NielsenE, KristensenJL, GajhedeM, et al (2012) Crystal structure of Lymnaea stagnalis AChBP complexed with the potent nAChR antagonist DH 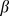 E suggests a unique mode of antagonism. PLoS One 7: e40757.2292790210.1371/journal.pone.0040757PMC3425559

[42] Law RJ, Henchman RH, McCammon JA (2005) A gating mechanism proposed from a simulation of a human alpha7 nicotinic acetylcholine receptor. Proc Natl Acad Sci USA 102: 6813–6818. 15857954 10.1073/pnas.0407739102 PMC1100735

[43] Cheng X, Ivanov I, Wang H, Sine SM, McCammon JA (2007) Nanosecond-timescale conformational dynamics of the human alpha7 nicotinic acetylcholine receptor. Biophys J 93: 2622–2634. 17573436 10.1529/biophysj.107.109843 PMC1989720

[44] Liu X, Xu Y, Li H, Wang X, Jiang H, et al (2008) Mechanics of Channel Gating of the Nicotinic Acetylcholine Receptor. PLoS Comput Biol 4: 0100–0110. 10.1371/journal.pcbi.0040019 PMC2211534 18225945

[45] Taly A, Delarue M, Grutter T, Nilges M, Novere NL, et al (2005) Normal Mode Analysis Suggests a Quaternary Twist Model for the Nicotinic Receptor Gating Mechanism. Biophys J 88: 3954–3965. 15805177 10.1529/biophysj.104.050229 PMC1305627

[46] Taly A, Corringer PJ, Grutter T, Prado L, Karplus M, et al (2006) Implications of the quaternary twist allosteric model for the physiology and pathology of nicotinic acetylcholine receptors. Proc Natl Acad Sci U S A 103: 16965–16970. 17077146 10.1073/pnas.0607477103 PMC1629088

[47] Cheng X, Lu B, Grant B, Law R, McCammon J (2006) Channel Opening Motion of alpha7 Nicotinic Acetylcholine Receptor as suggested by Normal Mode Analysis. J Mol Biol 355: 310–324. 16307758 10.1016/j.jmb.2005.10.039

[48] Samson AO, Levitt M (2008) Inhibition mechanism of the acetylcholine receptor by alpha-neurotoxins as revealed by normal-mode dynamics. Biochemistry 47: 4065–4070. 18327915 10.1021/bi702272j PMC2750825

[49] Zhou Y, Pearson JE, Auerbach A (2005) Phi value analysis of a linear, sequential reaction mechanism: theory and application to Ion Channel gating. Biophys J 89: 3680. 16183877 10.1529/biophysj.105.067215 PMC1366938

[50] Cheng X, Wang H, Grant B, Sine SM, McCammon JA (2006) Targeted molecular dynamics study of C-loop closure and channel gating in nicotinic receptors. PLoS Comput Biol 2: 1173–1184. 10.1371/journal.pcbi.0020134 PMC1584325 17009865

[51] Wang HL, Toghraee R, Papke D, Cheng XL, McCammon JA, et al (2009) Single-channel current through nicotinic receptor produced by closure of binding site C-loop. Biophys J 96: 3582–3590. 19413963 10.1016/j.bpj.2009.02.020 PMC2711404

[52] Gao F, Bren N, Burghardt TP, Hansen S, Henchman RH, et al (2005) Agonist-mediated conformational changes in Acetylcholine-binding Protein revealed by simulation and Intrinsic Tryptophan Fluorescence. J Biol Chem 280: 8443–8451. 15591050 10.1074/jbc.M412389200

[53] Amiri S, Sansom MS, Biggin PC (2007) Molecular dynamics studies of AChBP with nicotine and carbamylcholine: the role of water in the binding pocket. Protein Eng Des Sel 20: 353–359. 17595341 10.1093/protein/gzm029

[54] Shi C, Yu R, Shao S, Li Y (2008) Partial activatin of alpha7 nicotinic acetylcholine receptors: insights from molecular dynamics simulations. J Mol Model 105: 8280–8285. 10.1007/s00894-012-1618-6 23086458

[55] Yi M, Tjong H, Zhou HX (2008) Spontaneous conformational change and toxin binding in alpha7 acetylcholine receptor: Insight into channel activation and inhibition. Proc Nat Acad Sc 105: 8280–8285. 18541920 10.1073/pnas.0710530105 PMC2448828

[56] Liu X, Xu Y, Wang X, Barrantes FJ, Jiang H (2008) Unbinding of nicotine from the acetylcholine binding protein: steered molecular dynamics simulations. J Phys Chem B 112: 4087–4893. 18327929 10.1021/jp0716738

[57] Zhang D, Gullingsrud J, McCammon JA (2006) Potentials of Mean Force for Acetylcholine Unbinding from the alpha7 Nicotinic Acetylcholine Receptor Ligand Binding Domain. J Am Chem Soc 128: 3019–3026. 16506783 10.1021/ja057292u PMC2546508

[58] Maragliano L, Vanden-Eijnden E (2006) A temperature accelerated method for sampling free energy and determining reaction pathways in rare events simulations. Chem Phys Lett 426: 168–175.

[59] Ferrenberg AM, Alan M, Swendsen RH (1988) New Monte Carlo technique for studying phase transitions. Phys Rev Lett 61: 2635–2638. 10039183 10.1103/PhysRevLett.61.2635

[60] Maragliano L, Vanden-Eijnden E (2008) Single-sweep methods for free energy calculations. The Journal of Chemical Physics 128: 184110. 18532802 10.1063/1.2907241

[61] Maragliano L, Cottone G, Ciccotti G, Vanden-Eijnden E (2010) Mapping the network of pathways of CO diffusion in myoglobin. J Am Chem Soc 132: 1010–1017. 20039718 10.1021/ja905671x

[62] Sterpone F, Bonella S, Meloni S (2012) Early Stage of the Dehydrogenation of NaAlH4 by Ab Initio Rare Event Simulations. J Phys Chem C 116: 19636–19643.

[63] Monteferrante M, Bonella S, Meloni S, Vanden-Eijnden E, Ciccotti G (2008) Calculations of free energy barriers for local mechanisms of hydrogen diffusion in alanates. Scientific Modeling & Simulation SMNS 15: 187–189.

[64] Monteferrante M, Bonella S, Meloni S, Ciccotti G (2009) Modified Single Sweep Method for recostructing free energy landscape. Mol Sim 36: 1116.

[65] Ciccotti G, Meloni S (2011) Temperature accelerated Monte Carlo (TAMC): a method for sampling the free energy surface of non-analytical collective variables. Phys Chem Chem Phys 13: 5952–5959. 21340075 10.1039/c0cp01335h

[66] Lucid J, Meloni S, MacKernan D, Spohr E, Ciccotti G (2013) Probing the Structures of Hydrated Nafion in Different Morphologies Using Temperature Accelerated Molecular Dynamics simulations. J Phys Chem C 117: 774–782.

[67] Abrams CF, Vanden-Eijnden E (2012) On-the-fly free energy parameterization via temperature accelerated molecular dynamics. Chem Phys Lett 547: 114–119. 23226688 10.1016/j.cplett.2012.07.064 PMC3512107

[68] Abrams CF, Vanden-Eijnden E (2010) Large-scale conformational sampling of proteins using temperature-accelerated molecular dynamics. Proc Natl Acad Sci U S A 107: 4961–4966. 20194785 10.1073/pnas.0914540107 PMC2841907

[69] Vashisth H, Maragliano L, Abrams CF (2012) “DFG-flip” in the insulin receptor kinase is facilitated by a helical intermediate state of the activation loop. Biophys J 102: 1979–1987. 22768955 10.1016/j.bpj.2012.03.031 PMC3328698

[70] Vashisth H, Brooks CL (2012) Conformational sampling of maltose-transporter components in Cartesian collective variables is governed by the low-frequency normal modes. J Phys Chem Lett 3: 3379–3384. 23185650 10.1021/jz301650q PMC3505029

[71] Vashisth H, Skiniotis G, Brooks CL (2012) Using enhanced sampling and structural restraints to refine atomic structures into low-resolution electron microscopy maps. Structure 20: 1453–1462. 22958641 10.1016/j.str.2012.08.007 PMC3438525

[72] Vashisth H, Skiniotis G, Brooks CL (2013) Enhanced Sampling and Overfitting Analyses in Structural Refinement of Nucleic Acids into Electron Microscopy Maps. J Phys Chem B 117: 3738–3746. 23506287 10.1021/jp3126297 PMC3690198

[73] Vashisth H, Storaska AJ, Neubig RR, Brooks CL (2013) Conformational dynamics of a regulator of g-protein signaling protein reveals a mechanism of allosteric inhibition by a small molecule. ACS Chem Biol 8: 2778–2784. 24093330 10.1021/cb400568g PMC3876963

[74] NygaardR, ZouY, DrorRO, MildorfTJ, ArlowDH, et al (2013) The Dynamic Process of 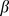 2-Adrenergic Receptor Activation. Cell 152: 532–542.2337434810.1016/j.cell.2013.01.008PMC3586676

[75] Scarpazza DP, Ierardi DJ, Lerer AK, Mackenzie KM, Pan AC, et al.. (2013) Extending the Generality of Molecular Dynamics Simulations on a Special-Purpose Machine. Parallel Distributed Processing (IPDPS). IEEE 27th International Symposium, 933–945 pp.

[76] Heinig M, Frishman D (2004) STRIDE: a web server for secondary structure assignment from known atomic coordinates of proteins. Nucleic Acids Res 32: W500–W502. 15215436 10.1093/nar/gkh429 PMC441567

[77] Edink E, Rucktooa P, Retra K, Akdemir A, Nahar T, et al (2011) Fragment growing induces conformational changes in Acetylcholine-Binding Protein: a structural and thermodynamic analysis. JACS 133: 5363–5371. 10.1021/ja110571r 21322593

[78] Akk G, Zhou M, Auerbach A (1999) A mutational analysis of the acetylcholine receptor channel transmitter binding site. Biophys J 76: 207–218. 9876135 10.1016/S0006-3495(99)77190-0 PMC1302512

[79] Terry AV Jr, Williamson R, Gattu M, Beach JW, McCurdy CR, et al (1998) Lobeline and structurally simplified analogs exhibit differential agonist activity and sensitivity to antagonist blockade when compared to nicotine. Proc Nat Acad Sc 37: 93–102. 10.1016/s0028-3908(97)00142-1 9680262

[80] Lape R, Colquhon D, Sivilotti LG (2008) On the nature of partial agonism in the nicotinic receptor superfamily. Nature 454: 722–728. 18633353 10.1038/nature07139 PMC2629928

[81] BillenB, SpurnyR, BramsM, van ElkR, Valera-KummerS, et al (2012) Molecular actions of smoking cessation drugs at 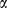 4 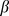 2 nicotinic receptors defined in crystal structures of a homologous binding protein. Proc Nat Acad Sc 109: 9173–9178.2261932810.1073/pnas.1116397109PMC3384148

[82] Zheng W, Auerbach A (2011) Decrypting the sequence of structural events during the gating transition of pentameric ligand-gated ion channels based on an interpolated elastic network model. PLoS Comput Biol 9: e1001046. domino. 10.1371/journal.pcbi.1001046 PMC3017109 21253563

[83] Mukhtasimova N, Free C, Sine SM (2005) Initial Coupling of Binding to Gating Mediated by Conserved Residues in the Muscle Nicotinic Receptor. J Gen Physiol 126: 23–39. 15955875 10.1085/jgp.200509283 PMC2266616

[84] Akk G, Zhou M, Auerbach A (1999) Initial coupling of binding to gating mediated by conserved residues in the muscle nicotinic receptor. Biophys J 76: 207–218.9876135

[85] Grosman C, Zhou M, Auerbach A (2000) Mapping the conformational wave of acetylcholine receptor channel gating. Nature 403: 773–776. 10693806 10.1038/35001586

[86] Purohit P, Mitra A, Auerbach A (2007) A stepwise mechanism for acetylcholine receptor channel gating. Nature 446: 930–933. 17443187 10.1038/nature05721

[87] Sali A, Blundell TL (1993) Comparative protein modelling by satisfaction of spatial restraints. J Mol Biol 234: 779–815. 8254673 10.1006/jmbi.1993.1626

[88] Eswar N, Webb B, Marti-Renom MA, Madhusudhan MS, Eramian D, et al (2006) Comparative Protein Structure Modeling using Modeller. Current Protocols in Bioinformatics 5: 5–6. 18428767 10.1002/0471250953.bi0506s15 PMC4186674

[89] Sander T, Bruun AT, Balle T (2010) Docking to flexible nicotinic acetylcholine receptors: a validation study using the acetylcholine binding protein. J Mol Graph Model 29: 415–424. 20884263 10.1016/j.jmgm.2010.08.004

[90] Kabsch W (1976) A solution for the best rotation to relate two sets of vectors. Acta Crystallographica 32: 922–923.

