# Supplementary figures and images for: Conformational Changes in Acetylcholine Binding Protein Investigated by Temperature Accelerated Molecular Dynamics
Source: PLoS One. 2014 Feb 13;9(2):e88555. doi: 10.1371/journal.pone.0088555 (PMC3923797; doi:10.1371/journal.pone.0088555)

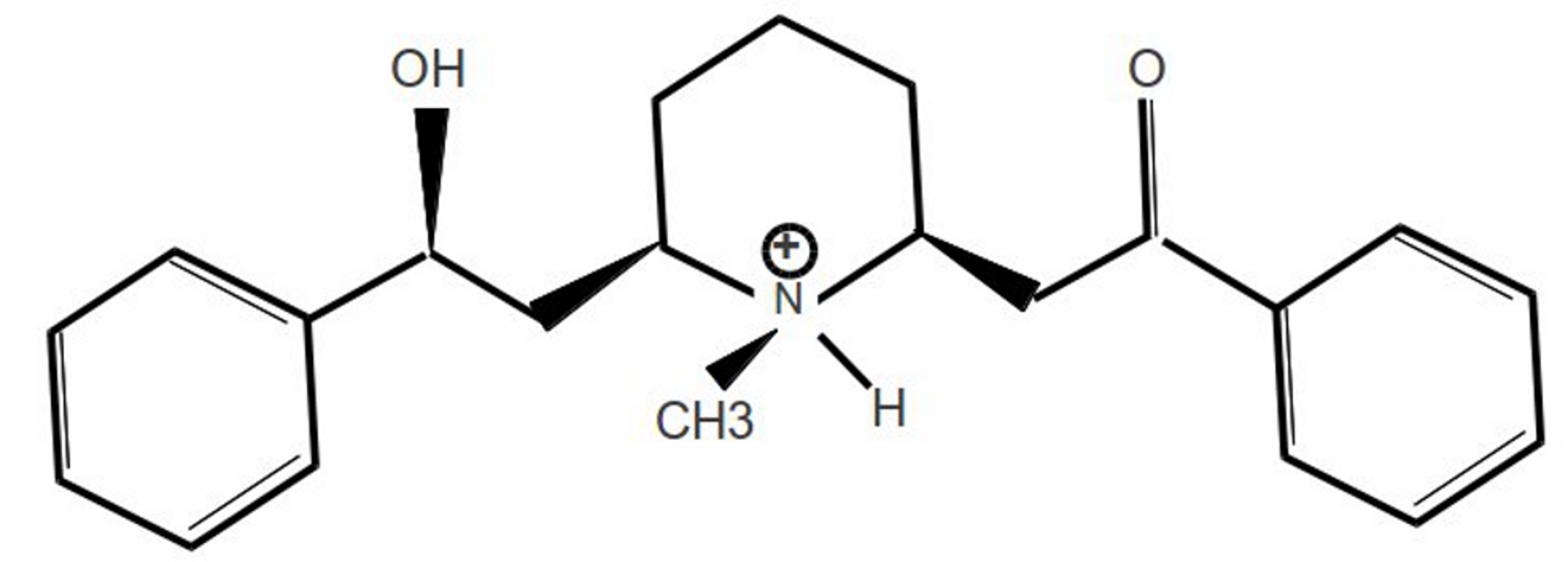

Supplement: Figure S1 — Structure of lobeline. Schematic structure of the lobeline molecule. (TIFF) [file pone.0088555.s001.tiff]

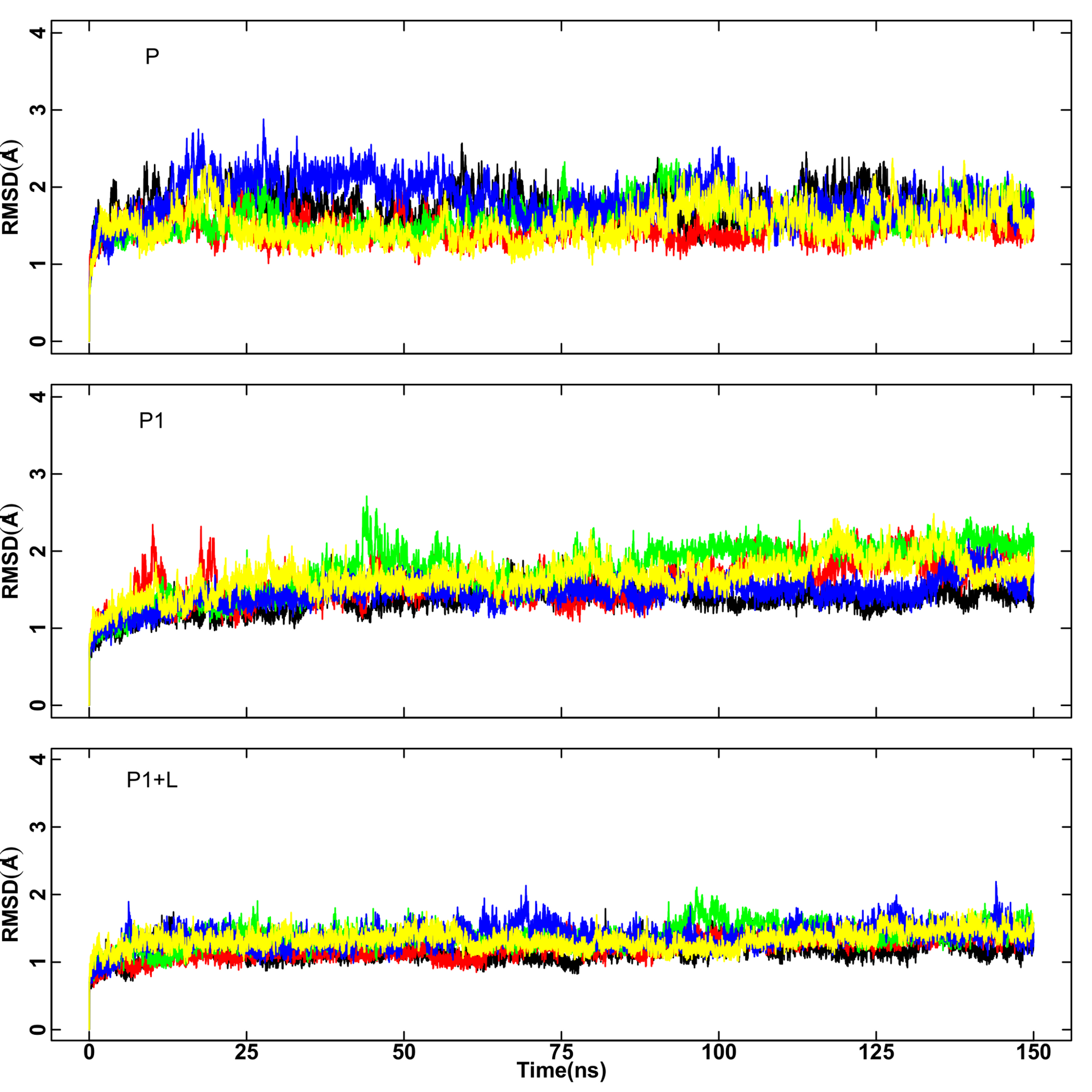

Supplement: Figure S2 — Root Mean Square Deviation of individual subunits. Root Mean Square Deviation (in Å) of individual subunits calculated from the starting conformations (after equilibration) as , where is the position of the atom at th time step, is the position of the atom in the reference structure, is the total number of atoms in the subunit. Upper panel: P; central panel: P1; lower panel: P1+L. The RMSD values are calculated after removing the roto-translational body motions of the single subunits [90]. The curves are colored according to the scheme in Fig. 1 in Main text. (TIFF) [file pone.0088555.s002.tiff]

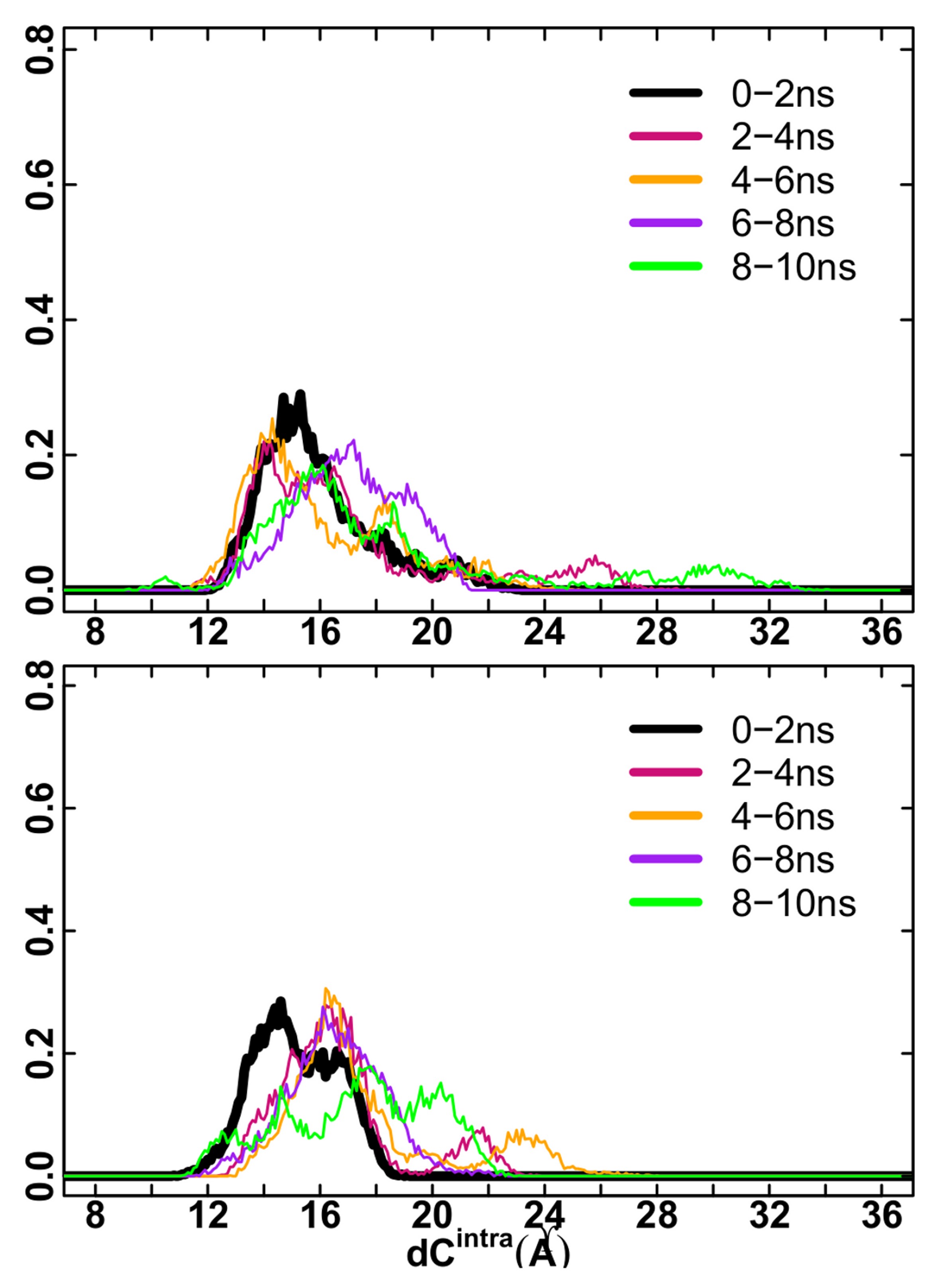

Supplement: Figure S3 — Distribution of values. Distribution of values (Å) calculated over the TAMD trajectory at different time slices (see legends), at = 10 kcal/mol. Upper panel: (P1)10; lower panel: (P1+L)10. (TIFF) [file pone.0088555.s003.tiff]

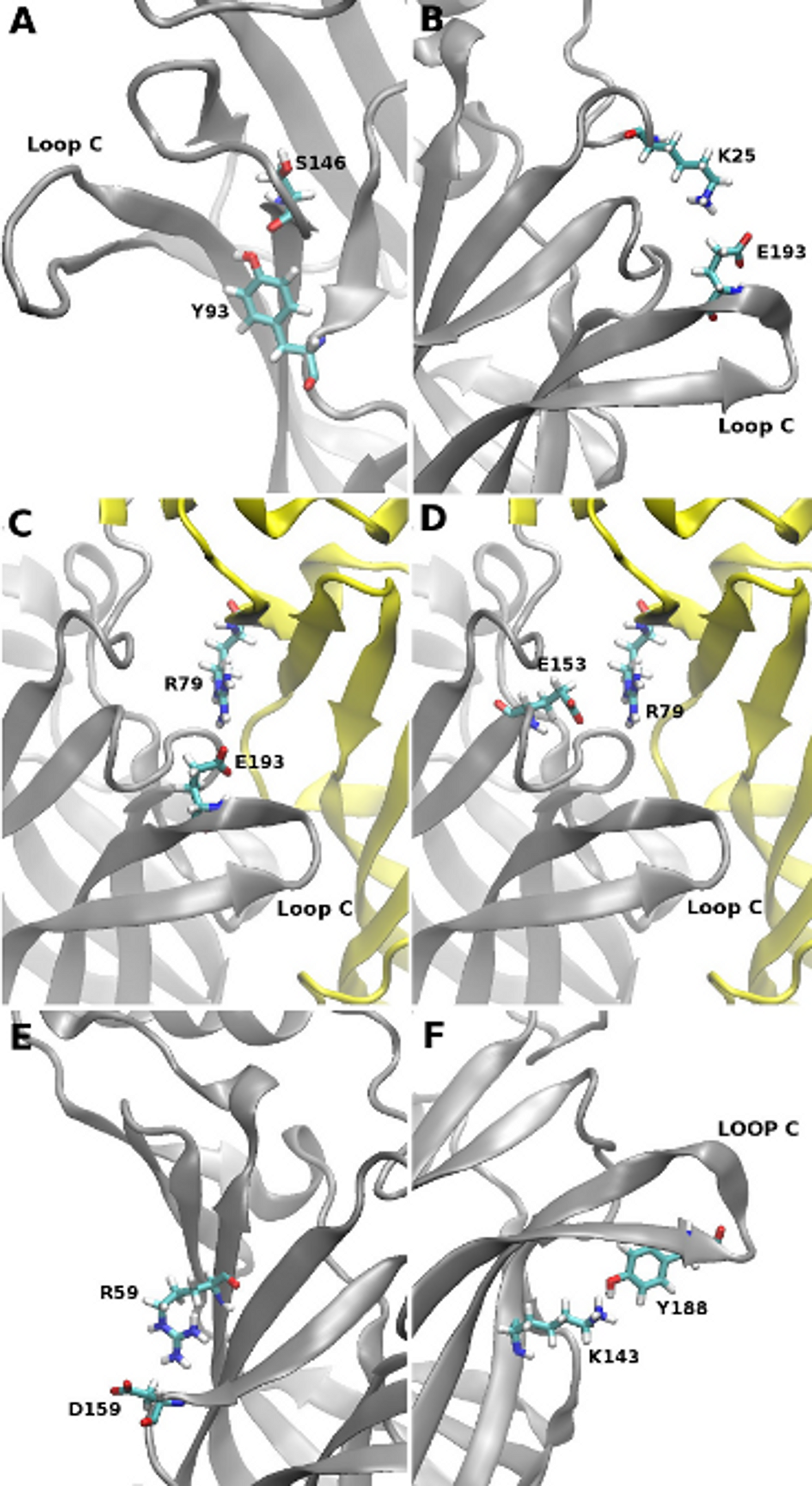

Supplement: Figure S4 — Protein residues involved in hydrogen bonds. Protein residues (in licorice) involved in hydrogen bonds analysed in this work. The protein is shown as a cartoon model. In panels C and D, the principal and complementary subunits are shown in gray and yellow, respectively. (TIFF) [file pone.0088555.s004.tiff]

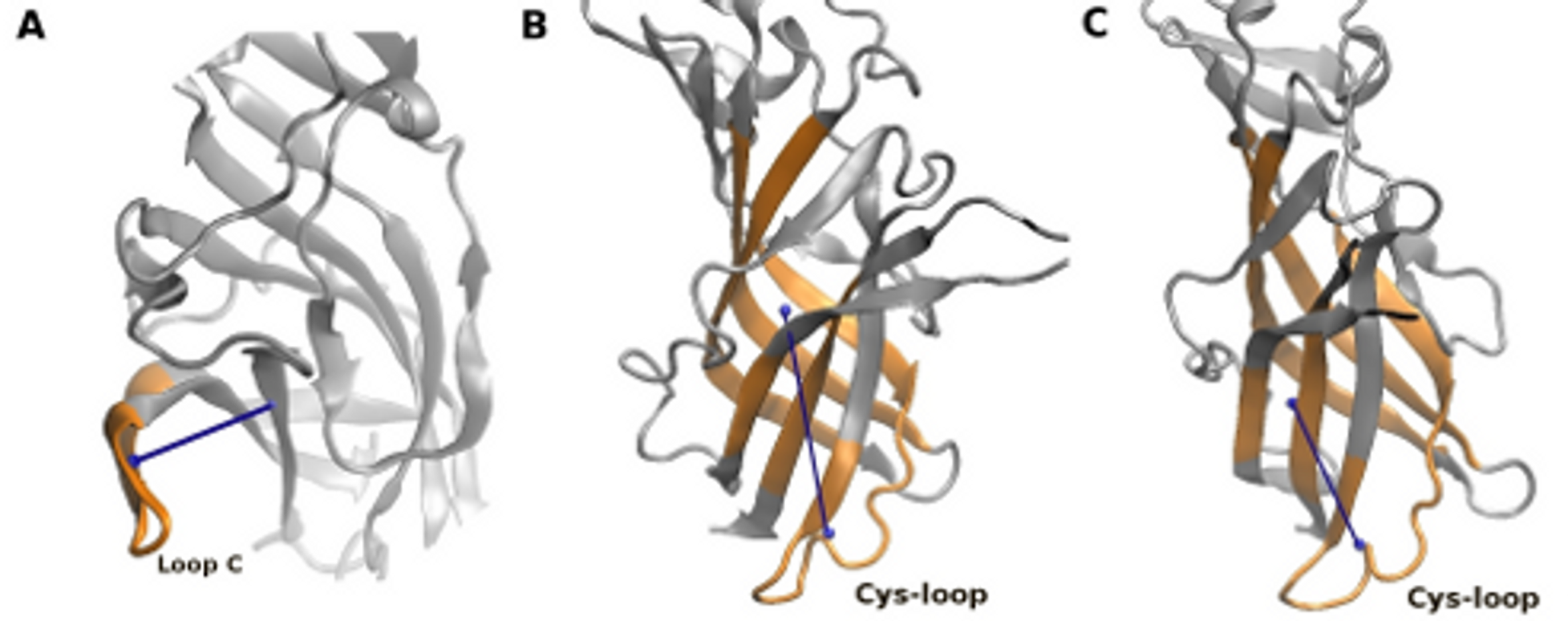

Supplement: Figure S5 — Schematic representation of the C-loop and cys-loop distances. Schematic representing of the C-loop and cys-loop distance parameters analyzed in this work. In A) the blue line indicates the ; B) ; C) . Protein is shown as a cartoon model. (TIFF) [file pone.0088555.s005.tiff]

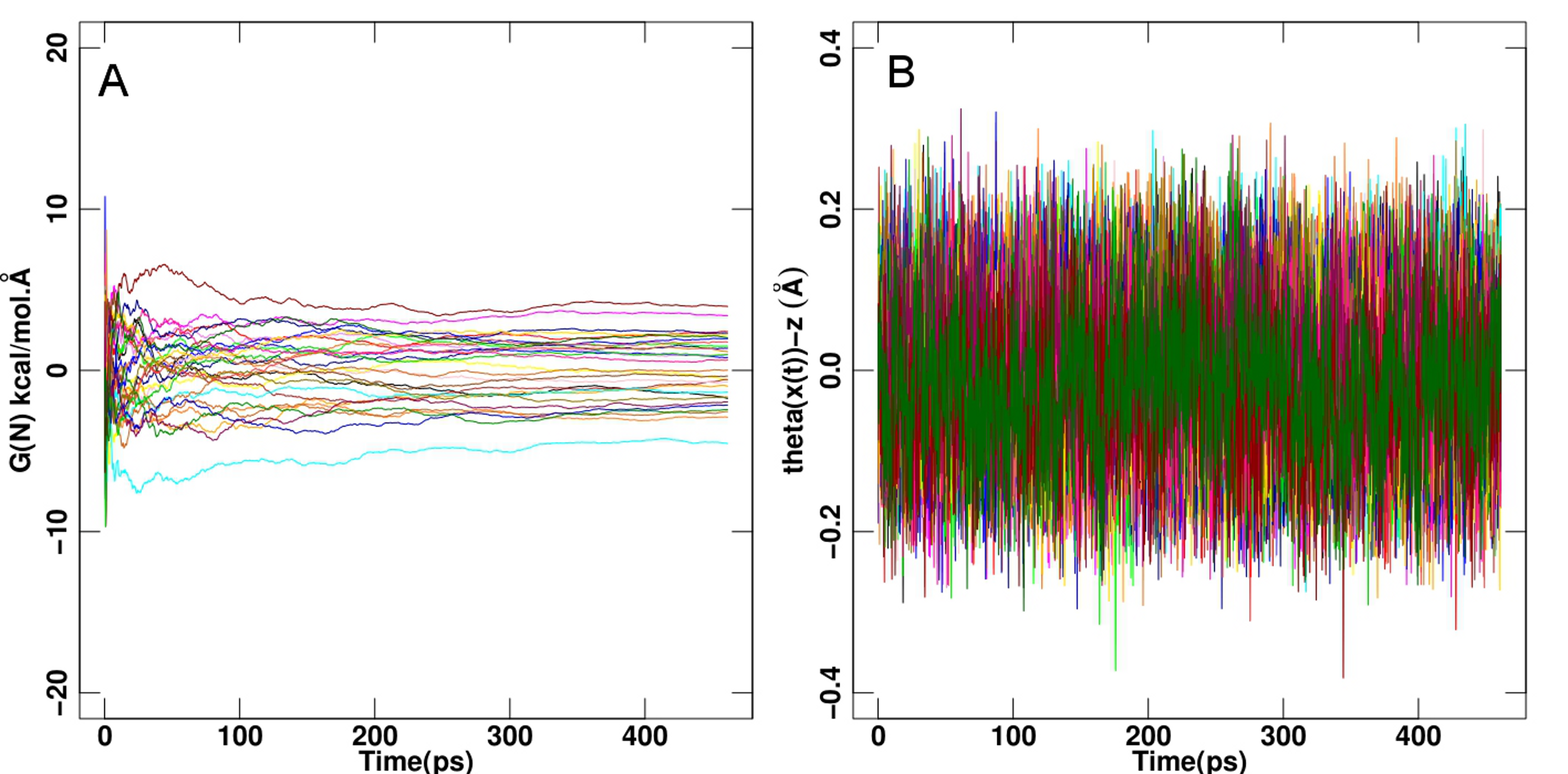

Supplement: Figure S6 — Running average of the restraining force for each CV and versus time. A) Running average of the restraining force for each CV, during MD simulation in which the CVs are fixed to their initial values; . B) values versus time for restrained dynamics, where is the total number of collective variables; . (TIFF) [file pone.0088555.s006.tiff]

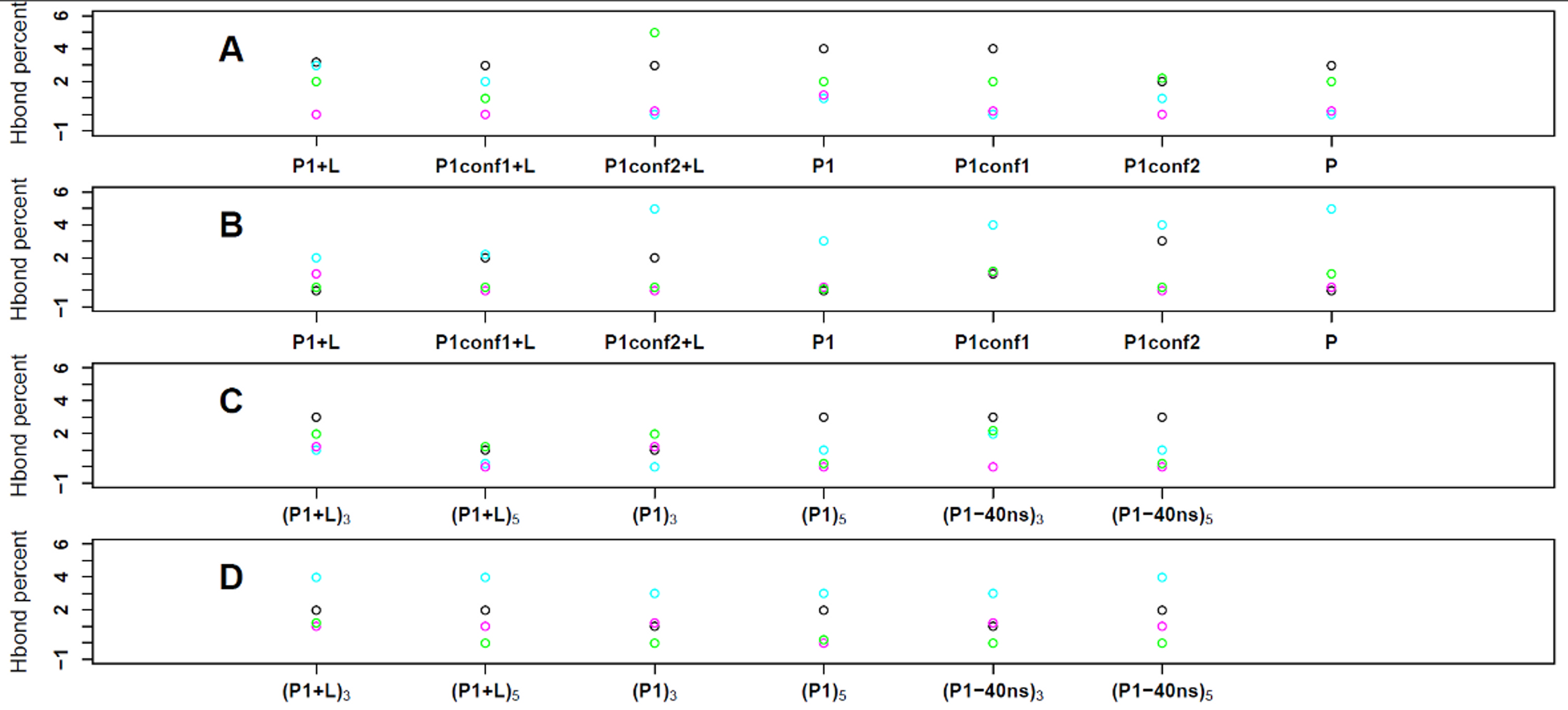

Supplement: Figure S7 — Hydrogen bond analysis. Number of subunits for which hydrogen bonds are present 50–90% of the time (A,C) or more than 90% of the time (B,D). The analyzed trajectories are MD (A,B) and TAMD at = 3 or 5 kcal/mol (C,D). The residue pairs are: ASP159/ARG59 (black), GLU153/ARG79 (cyan), GLU193/ARG79 (magenta), GLU193/LYS25 (green). The hydrogen bond is considered to be formed when the minimum distance among all possible donor/acceptor distances, for each pair, is less than 2.5 Å. (TIFF) [file pone.0088555.s007.tiff]
