# Supplementary material for: Conformational Changes in Acetylcholine Binding Protein Investigated by Temperature Accelerated Molecular Dynamics
Source: PLoS One. 2014 Feb 13;9(2):e88555. doi: 10.1371/journal.pone.0088555 (PMC3923797; doi:10.1371/journal.pone.0088555)
Supplement: Table S1 — Distances between lobeline and AChBP atoms. (PDF) [file pone.0088555.s011.pdf]

Table S1. Distances between lobeline and AChBP atoms.

Distance	P1+L	P1conf1+L	P1conf2+L
LOB-CO/TRP147-HE1	6.9 ± 0.6	6.6 ± 0.5	6.8 ± 0.6
LOB-NH/TRP147-CO	2.8 ± 0.7	4.1 ± 1.0	3.3 ± 0.6
LOB-OH/SER146-CO	2.5 ± 1.7	1.8 ± 0.2	1.8 ± 0.2
LOB-OH/TRP147-CO	3.8 ± 1.2	3.8 ± 0.7	3.5 ± 0.5
LOB-piperidine/TRP147-indole	4.9 ± 0.3	4.9 ± 0.3	4.9 ± 0.3
LOB-CH3/TYR188-ring	4.6 ± 0.7	4.7 ± 0.6	4.6 ± 0.7
LOB-CH3/TYR195-ring	3.7 ± 0.3	3.6 ± 0.3	3.6 ± 0.3

The values are obtained over the 150ns, 100ns and 100ns of MD simulations P1+L, P1conf1+L and P1conf2+L, respectively.
