# Supplementary material for: Conformational Changes in Acetylcholine Binding Protein Investigated by Temperature Accelerated Molecular Dynamics
Source: PLoS One. 2014 Feb 13;9(2):e88555. doi: 10.1371/journal.pone.0088555 (PMC3923797; doi:10.1371/journal.pone.0088555)
Supplement: Table S2 — Distances between lobeline and AChBP atoms. (PDF) [file pone.0088555.s012.pdf]

Table S2. Distances between lobeline and AChBP atoms.

Distance	$\overline{\beta}_3^{-1}$	$\overline{\beta}_5^{-1}$	$\overline{\beta}_7^{-1}$	$\overline{\beta}_{10}^{-1}$
LOB-CO/TRP147-HE1	6.9 ± 0.8	7.1 ± 0.9	7.3 ± 1.3	8.1 ± 3.3
LOB-NH/TRP147-CO	2.9 ± 0.7	2.8 ± 0.6	3.0 ± 1.2	5.1 ± 6.1
LOB-OH/SER146-CO	1.9 ± 1.0	2.5 ± 1.7	3.6 ± 2.2	6.3 ± 5.3
LOB-OH/TRP147-CO	3.4 ± 1.1	3.9 ± 1.3	4.7 ± 1.7	7.2 ± 5.7
LOB-piperidine/TRP147-indole	4.9 ± 0.3	5.0 ± 0.4	5.1 ± 0.8	6.3 ± 4.0
LOB-CH3/TYR188-ring	4.6 ± 0.7	7.6 ± 3.4	10.0 ± 4.5	13.7 ± 7.2
LOB-CH3/TYR195-ring	3.7 ± 0.4	4.7 ± 1.9	5.6 ± 2.7	10.1 ± 5.6

The values are obtained over the TAMD simulations with $\overline{\beta}^{-1}$ equal to 3, 5, 7 and 10 kcal/mol, started from the configuration at 40ns along the unbiased MD P1+L simulation. Lengths of the runs were 20ns, 30ns, 20ns and 10ns respectively.
