# Supplementary material for: Conformational Changes in Acetylcholine Binding Protein Investigated by Temperature Accelerated Molecular Dynamics
Source: PLoS One. 2014 Feb 13;9(2):e88555. doi: 10.1371/journal.pone.0088555 (PMC3923797; doi:10.1371/journal.pone.0088555)
Supplement: Table S3 — Size and contents of the simulated systems. (PDF) [file pone.0088555.s013.pdf]

Table S3. Size and contents of the simulated systems.

Name	Number of atoms	Water box size (Å)	Counter-ions
P1+L	114578	103.7, 103.7, 103.7	45 Na^+
P1	114360	103.7, 103.7,103.7	50 Na^+
P	116043	107.6, 108.4, 96.7	50 Na^+
P1conf1+L	114782	103.8, 103.8, 103.8	45 Na^+
P1conf2+L	114758	103.8, 103.8, 103.8	45 Na^+
P1conf1	114564	103.8, 103.8, 103.8	50 Na^+
P1conf2	114537	103.8, 103.8, 103.8	50 Na^+

Size and contents of the simulated systems.
