# Supplementary material for: Conformational Changes in Acetylcholine Binding Protein Investigated by Temperature Accelerated Molecular Dynamics
Source: PLoS One. 2014 Feb 13;9(2):e88555. doi: 10.1371/journal.pone.0088555 (PMC3923797; doi:10.1371/journal.pone.0088555)
Supplement: Text S1 — (PDF) [file pone.0088555.s014.pdf]

Conformational changes in acetylcholine binding protein investigated by Temperature Accelerated Molecular Dynamics Supplementary Material

New AChBP models

In order to create alternative starting points for the lobeline-bound AChBP structure, we modified the residues for which alternative sidechain conformations were visible in 2BYS (holo state): ARG59, ARG97, GLN105, GLN186 and ARG206. In order to alter the conformations of these residues and to favor the AChBP transition toward its apo state, we replaced the sidechain conformations by the most populated sidechain conformations observed for the same residues among 2W8E (apo state) subunits. As these new configurations are chosen as the ones experimentally observed in the apo AChBP, they carry relevant information concerning the behavior of AChBP in the absence of ligand. In this way we obtained the AChBP conformation we label conf1. An additional altered starting conformation, conf2, was produced by adding to the previously described modifications the one obtained by replacing the LYS-143 sidechain conformation in 2BYS by the conformation observed in 2W8E. This additional starting point was introduced because the mutation αK145A in the α subunit of nAChRs profoundly impairs receptor activation [1]. By modifying these residues configurations into the ones corresponding to the apo state of AChBP, we see that AChBP get closer to the apo state, by monitoring the C-loop behavior. Thus, the relevance of these residues modifications is supported by the plausible effects the modifications have on the ACHBP global behavior.

Molecular Dynamics simulations

MD simulations were run with the NAMD program NAMD 2.7b2 [2], with the the force field CHARMM27. In TAMD runs, the atomic force coming from the coupling potential and the evolution of the CVs are implemented via a Tcl script linked to the NAMD code.

Topology for the lobeline (Fig. S1) was generated by Antechamber [3]; ligand partial atomic charges were obtained by us from *ab initio* calculations with Gaussian 03 [4]. The pyrrolidine nitrogen on the lobeline was assumed to be protonated. Lobeline parameters are also given as Supplementary Material, see file "Lobeline_parameters".

Each protein was solvated in a cubic box of TIP3P [5] water molecules; to ensure the neutrality of the box, 45 or 50 water molecules were then replaced by 45 or 50 sodium counter-ions, in the ligand bound and apo protein systems, respectively (see Table S3 for details on the simulation set-up).

Each solvated system underwent the following equilibration steps: a) five rounds of 1000 steps minimization with position restraints on the backbone atoms, by decreasing the restraints at each round (force constant from 5 kcal $\cdot$ mol^{-1}Å^{-2} to 1 kcal $\cdot$ mol^{-1}Å^{-2}); b) gradual temperature increase from 25 to 325 K in 2400 steps, with restraints on the backbone heavy atoms (force constant equal to 1 kcal $\cdot$ mol^{-1}Å^{-2}) and by using Langevin dynamics (friction constant 2ps^{-1}); c) after removing the restraints, the system was equilibrated over 2ns at constant pressure (1bar) and temperature (T=300K) by using the Langevin-Hoover pressure control method as implemented in NAMD [6].

The simulations for data collection (after minimization and equilibration) were then performed at constant temperature of 300K by using Langevin dynamics (friction constant 2ps^{-1}). Periodic boundary conditions were used [7]; van der Waals interactions have been cut off beyond a distance of 12 Å. Electrostatic interactions were calculated by the Ewald sum using the PME method [8]. Chemical bonds involving hydrogens were kept fixed using the SHAKE constraint algorithm [9], with a 1fs time step. Coordinate sets were saved every 1ps for data analysis.

Determining the value of fictitious friction $\overline{\gamma}$ and spring constant k

In the TAMD equations, $\overline{\gamma}$ must be high enough to let the Cartesian variables equilibrate at a fixed value of the CV. We can estimate the adequate value of $\overline{\gamma}$ by computing the running average $G_j(N)$, with N the total number of time-steps, of the restraining force for each CV during an MD simulation in which the CVs are fixed at their initial values, and evaluating the time it takes for the running average to reach convergence,

$$G_j(N) = \frac{k}{N}\sum_{i=1}^{N}\left[\theta_j(x(t)) - z_j\right]. \tag{1}$$

where$j$ is the number of CVs. If we call this time τ, then $\overline{\gamma}$ must be of the order of τ^{-1}. The mass center coordinates of loop C and Cys loop for each subunit are considered as CVs to be accelerated (for a detailed discussion on the CV's chosen, see also in "Materials and Methods" in the Main text). In the pentamer, the total number of CVs is then 30. In Fig.S6A $G_j(N)$ is plotted for all the CVs; a friction coefficient of $200ps^{-1}$ is enough to allow accurate estimate of the local free-energy gradients.

The value of the spring constant k should be sufficiently large such that $\theta_j(x(t)) \approx z_j$ but not so large to introduce numerical instabilities (less than the k's in the covalent bond terms of CHARMM force field). In Fig.S6B, the values of $\theta_j(x(t)) - z_j$ are plotted versus time for the restrained dynamics of CVs by using $k = 100\frac{kcal}{mol\mathring{A}^2}$; the fluctuations are less than 0.4 Å , small enough to keep the values of $\theta_j(x(t))$ close to z_j. $k = 100\frac{kcal}{mol\mathring{A}^2}$ was then used in the TAMD simulations.

Hydrogen bond analysis

At each frame of P1, P1+L and P MD simulations, all possible distances between protein donor and acceptor groups located at more than 10 residues in the protein sequence, have been calculated. Statistics on the whole trajectories, for individual subunits, were then performed: we selected the donor/acceptor distances found smaller than 2.5 Å and present in more than 80% along at least one trajectory and less than 20% along at least one among other trajectories. According to this criterion, we selected the residues: SER146/TYR93, GLU193/LYS25, establishing intra-subunit hydrogen bonds, and GLU193/ARG79, GLU153/ARG79, establishing hydrogen bonds between principal and complementary subunits (see Fig.S4). In addition, hydrogen bonding in the pairs ASP159/ARG59 and LYS143/TYR188 was analyzed. The importance of these residues is confirmed, except for LYS25 and GLU153, by their presence in the same position along the sequence in AChBP species and in nAChRs [10]. Results on the LYS143/TYR188 and SER146/TYR93 pairs are fully described in the Main text; as for the other pairs, results are described in the next section.

Hydrogen bonds between some specific residues: the case of GLU193/LYS25, GLU193/ARG79, GLU153/ARG79, ASP159/ARG59.

At variance with the hydrogen bond formed by SER146/TYR93 and LYS143/TYR88, those involving residues pairs: GLU193/LYS25, GLU193/ARG79, GLU153/ARG79, ASP159/ARG59, do not display a global behavior different among the various MD and TAMD trajectories at $\bar{\beta} = 3$ or 5 kcal/mol. Indeed, hydrogen bonds located in individual subunits show large variations in the percentages of formation, but, the number of subunits for which a given hydrogen bond is established in the 50-90% (Fig. S7, A,C) and in more than 90% (Fig. S7, B, D) of the simulation length, does not strongly vary among MD and TAMD

trajectories. The GLU193/LYS25 hydrogen bond is mostly formed in more than 2 subunits but only for a percentage of time in the 50-90% range (green symbols in Fig. S7A). The GLU193/ASP79 hydrogen bond is formed usually only in one subunit, but is quite often established for more than 90% of the simulation length (magenta symbols in Fig. S7B). The hydrogen bond GLU153/ARG79, between principal and complementary subunit, is the most stable hydrogen bond, mostly formed in more than 90% of the time in the whole pentamer (cyan symbols in Fig. 6B). The hydrogen bond ASP159/ARG59 is slightly less stable: it is formed in most of the subunits but in the 50-90% range (black symbols in Fig. S6A). The set of hydrogen bonds described here forms a network connecting the residues: LYS25, GLU193, GLU153 from the principal subunit along with ARG79 and ASP159 from the complementary subunit, helping in that way to maintain the global architecture of the orthosteric site. The strongest hydrogen bond GLU153/ARG79 is specially important to keep together the two subunits. Similar hydrogen bond stabilities are observed in MD and TAMD trajectories, which supports the ability of TAMD at $\bar{\beta} = 3$ or 5 kcal/mol to preserve the architecture of the orthosteric site.

TAMD exploration and the role of SER146/TYR93 bond

Results reported in the Main text suggested how the lobeline-bound to unbound transition could develop through the formation/disruption of few key hydrogen bonds. As showed in the following analysis, the SER146/TYR93 bond in particular fully discriminates between the two end-state conformations, providing a structural characterization of the bound-to-unbound path as explored by TAMD.

As pointed out by the d_C^{intra} distributions, shown in Fig.3 in the Main text, the natural apo P state of AChBP protein is indeed a combination of two different states; let we call P/1 the state in which the monomer has closed C-loop (e.g. as in a P1+L monomer), and P/2 the state in which the monomer has opened C loop. The latter state is the most populated in the P stationary trajectory. We measured along the TAMD trajectories the all atoms Root Mean Square Deviation (RMSD) of SER146 and TYR93 with respect to the reference states P/1, P/2, P1+L. The probability to obtain a RMSD value less than 2 Å from each reference state was calculated, as well as the probability to obtain a RMSD value less than 2 Å from two different reference states at the same time. Results from the TAMD at $\overline{\beta}^{-1} = 3$ kcal/mol, starting from both the P1 and P1-40ns initial conditions, are shown in Fig.S8. Results from the TAMD at $\overline{\beta}^{-1} = 5$, 7 and 10 kcal/mol are shown in Fig.S9; results from the TAMD simulations in the presence of lobeline are shown in Fig. S10.

In Fig. S8, we observe that the probability to obtain a RMSD value less than 2 Å from both P/1 and P1+L at the same time is zero. This means that the relative position of SER146 and TYR93 in the P/1 state of P and P1+L is really different, although the C-loop is closed in both of them. Moreover results show how TAMD was efficient in exploring also the P/2 state, already at $\overline{\beta}^{-1} = 3$ kcal/mol. At higher fictitious energies (Fig.S9), TAMD evolves the system to states distinct from the natural apo state P, as shown by the higher probability of RMSD value less than 2 Å from none of the reference states considered. This was already observed in the d_C^{intra} distributions shown in Fig.4 in the Main text. The presence of the lobeline fully prevents the transition (Fig.S10); the system could explore both the P/1 and P/2 conformations only at very high fictitious energies (greater than 5 kcal/mol).

References

1. Mukhtasimova N, Free C, Sine SM (2005) Initial Coupling of Binding to Gating Mediated by Conserved Residues in the Muscle Nicotinic Receptor. J Gen Physiol 126: 23-39.

2. Phillips JC, Braun R, Wang W, Gumbart J, Tajkhorshid E, et al. (2005) Scalable molecular dynamics with NAMD. J Comput Chem 26: 1781-1802.

3. Wang J, Wang W, Kollmann P, Case D (2005) Antechamber, An Accessory Software PackageFor Molecular Mechanical Calculation. J Comput Chem 25: 1157-1174.

4. Frisch MJ, Trucks GW, Schlegel HB, Scuseria GE, Robb MA, et al. (Gaussian, Inc., Pittsburgh PA, 2003) Gaussian 03, Revision B.02 .

5. Jorgensen W, Chandrasekhar J, Madura J, Impey R, Klein M (1983) Comparison of simple potential functions for simulating liquid water. J Chem Phys 79: 926-935.

6. Feller SE, Zhang Y, Pastor RW, Brooks BR (1995) Constant pressure molecular dynamics simulation: The Langevin piston method. J Chem Phys 103: 4613.

7. Allen MP, Tildesley DJ (1989) Computer Simulation of Liquids. Oxford Science publications. Oxford University Press, USA.

8. Darden T, York D, Pedersen L (1993) Particle Mesh Ewald and an N.log(N) method for Ewald sums in large systems. J Chem Phys 98: 3684-90.

9. Ryckaert JP, Ciccotti G, Berendsen HJC (1977) Numerical integration of the cartesian equations of motion of a system with constraints and Molecular dynamics of n-alkanes. J Comput Phys 23: 327-341.

10. Hansen SB, Sulzenbacher G, Huxford T, Marchot P, Taylor P, et al. (2005) Structures of Aplysia AChBP Complexes with Nicotinic Agonists and Antagonists Reveal Distinctive Binding Interfaces and Conformations. EMBO J 24: 2625-3646.
